# Effects of Radishes, Apples, and Pears on the Lactic Acid Bacteria and Nutritional and Functional Qualities of Flavored Soy Sauce

**DOI:** 10.3390/foods9111562

**Published:** 2020-10-28

**Authors:** Ashutosh Bahuguna, Il Guk Jo, Jong Suk Lee, Myunghee Kim

**Affiliations:** 1 Department of Food Science and Technology, Yeungnam University, Gyeongsan 38541, Korea; ashubahuguna@ynu.ac.kr (A.B.); 11nation@naver.com (I.G.J.); 2 Division of Food & Nutrition and Cook, Taegu Science University, Daegu 41453, Korea; jslee1213@ynu.ac.kr; 3 Research Institute of Cell Culture, Yeungnam University, Gyeongsan 38541, Korea

**Keywords:** cellular antioxidant, fermentation, flavored soy sauce, free amino acids, GC-MS, lactic acid bacteria, *Tetragenococcus halophilus*

## Abstract

Producers of soy sauce are constantly making efforts to improve the sensory quality and nutritional value of their products. In this study, radishes, apples, and pears were used to prepare a distinctly flavored soy sauce, and the lactic acid bacteria, volatile compound content, and nutritional and functional qualities of the product were compared with two commercial flavored soy sauce products. Comparable physiochemical properties, antioxidant activities (in vitro and cellular), and higher prevalence of lactic acid bacteria (7.74 ± 0.55 log CFU mL^−1^) were observed in the prepared flavored soy sauce than in commercial flavored soy sauce. The comprehensive enzyme activity profile of the isolated lactic acid bacteria, *Tetragenococcus halophilus* (NCBI GenBank Accession no. MN270899), revealed the absence of any harmful enzymes such as β-glucuronidase. Moreover, the cell-free extract of *T. halophilus* showed xanthine oxidase inhibitory activity (half maximal inhibitory concentration (IC_50_) = 0.79 mg mL^−1^), suggesting that the product possessed functionality against xanthine oxidase-induced oxidative stress. Additionally, the prepared flavored soy sauce had higher amounts of total free amino acids (48.68 mg mL^−1^) and organic acids (7.77 mg mL^−1^). These results suggest that radishes, apples, and pears at a defined ratio are suitable for the large scale production of a flavored soy sauce with improved nutritional and functional qualities.

## 1. Introduction

Soybeans (*Glycine max*), which are a rich source of nutrients, are consumed whole or in the form of fermented products, such as Korean *gochujang* (a red chilli paste), *doenjang* (a brined soybean paste), *chungukjang* (a seasoning paste), and *kanjang* (soy sauce) [1,2], Japanese *miso* (a seasoning paste), and Indonesian *tempeh* (a compressed soybean cake) [3]. In particular, dark-colored salty soy sauces are the most popular fermented products in East Asian countries and are gaining acceptability worldwide due to their unique tastes, aromas, and health benefits [4]. Soy sauce contains various bioactive components exhibiting numerous properties such as anti-carcinogenic, antimicrobial, antiplatelet, and immunomodulating activity [5].

The preparation of soy sauce differs between regions based on the selection of raw materials, microbial starter cultures, strength of the brine, and time and temperature of the fermentation process, which eventually forms soy sauce with distinct properties [6]. In Korea, soy sauce is prepared by either the traditional method or the industrial method. In the traditional method, boiled soybeans are used to produce *meju*, with the fermentation being carried out by naturally evolved microorganisms. By contrast, the industrial method uses defatted soybeans and wheat flour to produce the *meju*, with the inoculation of *Aspergillus oryzae* and *Bacillus* species [7]. Further, in both processes, *meju* is soaked in the brine solution and fermented for several months to create the soy sauce [8]. Soy sauce prepared in the traditional way is generally preferred by consumers [7,9], due to the unique taste and aroma imparted by the activities of a wide variety of naturally occurring molds (*Aspergillus flavus*, *A. fumigatus*, *A. oryzae*, *Mucor adundans*, *Rhizopus nigricans*, *R. oryzae*, and *R. stolonifer*), yeasts (*Candida edax*, *Saccharomyces cerevisiae*, and *S. kluyveri*), and bacteria (*Bacillus citreus*, *B. circulans*, *B. licheniformis*, *B. megaterium*, *B. mesentericus*, and *Lactobacillus* species) [8].

The high usage of soy sauce urges companies to enhance the quality in terms of sensory and nutrition. Most of the recent studies have focused on the development of new microbial strains and the optimization of the fermentation process to improve the production and functionalities of soy sauces [10]. Along with these techniques, the inclusion of other ingredients is emerging as a new trend for developing novel soy sauces with better sensory and functional attributes.

Apples, pears, and radishes are rich sources of dietary supplements and have unique tastes and aromas. They contain important phytochemicals, such as carotenoids, flavonoids, isoflavonoids, and phenolic acids, which have many beneficial effects on human health [11,12,13]. Apples, in particular, are extensively consumed worldwide and have been linked in epidemiological studies to a decreased risk of diabetes, asthma, and cardiovascular diseases [12].

In this study, several flavored soy sauce products were prepared at laboratory scale by adding mixtures of radish, apple, and pear (at various proportions) and examined for quality. The best combination was then selected for production of the flavored soy sauce at plant scale (hereinafter PFSS) in order to evaluate the feasibility of its mass-level production and to compare with the two most popular commercial Korean flavored soy sauce products (hereinafter CFSS-A and CFSS-B). 

## 2. Materials and Methods

### 2.1. Chemicals and Reagents

The soybeans (Korean origin, Daewon cultivar) were purchased from a local market in Cheongsong (36°26′ N, 129°03′ E), Gyeongsangbuk-do, Republic of Korea. The apples (Hongro cultivar), radishes (Palkwang cultivar), and pears (Singo pear cultivar) were purchased from local farms in Gyeongsan (35°49′ N, 128°44′ E), Gyeongsangbuk-do, Republic of Korea. Salt (Sinan, Jeollanam-do, Republic of Korea) was purchased from a supermarket in Gyeongsan. Standard sugars and sugar alcohols (sucrose, glucose, galactose, fructose, mannitol, and sorbitol) and organic acids (lactic acid, acetic acid, and succinic acids) were purchased from Sigma-Aldrich (St. Louis, MO, USA). The high-performance liquid chromatography (HPLC)-grade solvents were purchased from Merck (Darmstadt, Germany). Unless otherwise stated, all other chemicals used were of the highest quality and used as supplied.

### 2.2. Preparation of Flavored Soy Sauces at Laboratory Scale

**Figure 1:**
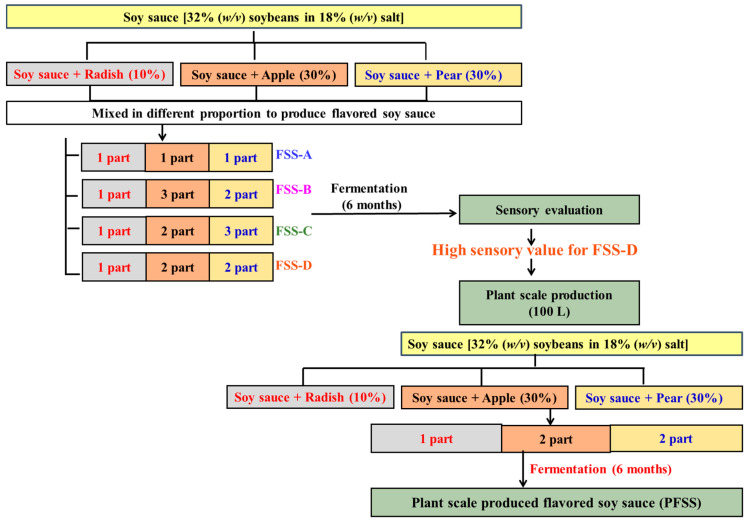
Sequential steps representing the preparation of different flavored soy sauce (FSS-A, FSS-B, FSS-C, and FSS-D) at laboratory scale and preparation of flavored soy sauce at plant scale (PFSS).

The traditional Korean soy sauce was prepared with 32% soybeans *meju* in 18% salt solution and served as control. For the *meju* preparation, boiled soybeans were crushed and transferred into stainless steel square plates to make the soybean blocks and fermented at ambient temperature for 2 months by natural microflora. Source materials for the preparation of flavored soy sauce, such as pulverized radish, apple, and pear, were supplemented separately with the basic ingredients of traditional Korean soy sauce at 10, 30, and 30% proportion (*w/w*), respectively. Radish, apple, and pear were pulverized using the mechanical grinder. Radish, apple, and pear-supplemented soy sauces were further mixed in different proportion and naturally fermented for 6 months to produce 4 distinct types of flavored soy sauce (designated FSS-A through FSS-D; Table 1) (Figure 1 and Supplementary Materials S1.1). All the experiments were at least carried out in triplicate.

The selection of 10, 30, and 30% proportion of radish, apple, and pear for the preparation of flavored soy sauce was based on the preliminary sensory study performed with different proportions (0, 10, 30, and 50%) of these vegetable and fruits to supplement soy sauce. In the sensory study, the highest preference was accredited for 10% radish and 30% for each apple and pear supplement soy sauce (Supplementary Figure S1). Therefore, 10, 30, and 30% proportion of radish, apple, and pear were selected for the final preparation of flavored soy sauce.

### 2.3. Determination of the pH, Total Soluble Solids, and Browning Potential of the Flavored Soy Sauces

The pH of the flavored soy sauce samples was evaluated monthly throughout the fermentation process (0–6 months) using a pre-calibrated pH meter (Thermo Electron Corporation; Beverly, MA, USA). The content of total soluble solids was measured using a refractometer (Brinkmann Instruments Inc.; Rockaway, NJ, USA) after removal of the solid particles by centrifugation at 8000× *g*. The 0.2 µm pore size membrane-filtered, 10-fold diluted flavored soy sauce samples (0–6 months) were subjected to a browning analysis by measuring the absorbance at 420 nm in a spectrophotometer (Tecan; Mannedorf, Switzerland).

### 2.4. Sensory Evaluation

Sensory evaluation with respect to flavor, salty taste, sweetness, and overall acceptability was examined by a panel of twenty trained people belonging to different age groups. The selection of panelists was made based on their sensitivity towards the 4 tastes (sweet, salty, bitter, and sour). The panelists have experience and sufficient expertise in conducting sensory and descriptive analysis. Before starting the analysis, panelists discussed the taste properties of the food samples. Plain bread and drinking water were served to each panelist to rinse the mouth while moving from one sample to another. Flavored soy sauce was served to each panelist in a randomized manner. Sensory evaluation for each parameter was judged on a 7 point scale [14]. The sensory score of numerical value 1 represents extreme dislike while the maximum numerical value 7 represents extreme like of the sample. 

### 2.5. Preparation of the Flavored Soy Sauce at Plant Scale 

**Figure 2:**
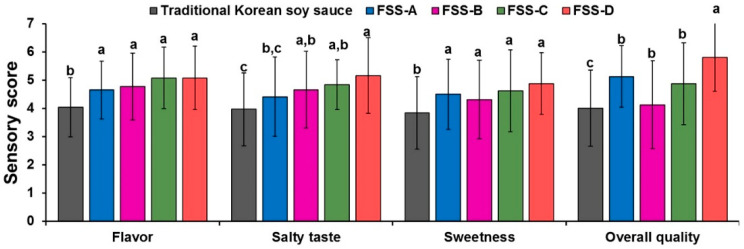
Sensory characteristics of 4 different flavored soy sauces after 6 months of fermentation. Each value represents mean ± SD (*n* = 20). Sensory characteristics were judged on a 7 point scale (7: extremely like, 1: extremely dislike). Different lowercase letters on a bar indicate significantly statistical differences (*p* < 0.05).

The best combination of flavored soy sauce obtained from the laboratory scale preparations was selected for plant scale production in a 100 L porcelain jar (Figure 1) (Supplementary Materials S1.1). The selection of the best flavored soy sauce preparation was based on the sensory qualities (Figure 2) and physiochemical parameters. Plant scale flavored soy sauce fermentation was carried out for 6 months. Simultaneously, a traditional Korean soy sauce without any addition of radish, pear, and apple served as the control, while the two most popular commercial Korean flavored soy sauces, named as CFSS-A and CFSS-B (Supplementary Materials S1.2), were used for the comparative assessment with respect to physicochemical properties (pH, total soluble solids, browning potential), amino nitrogen, sugars, organic acids, free amino acids, volatile compounds, antioxidants (in vitro and cellular), and presence of lactic acid bacteria.

### 2.6. Determination of Amino Nitrogen, Free Sugars, Organic Acids, and Amino Acids

Amino nitrogen was estimated using the formol titration method [15]. In brief, 0.5 mL of the flavored soy sauce sample was diluted 20 times with distilled water and shaken for 30 min. Then, 20 mL of 35% formalin was added, and the mixture was titrated against 0.1 N NaOH up to pH 8.5.

Quantification of the free sugars and organic acids was obtained by HPLC analysis [16]. For the analysis of the free sugars and organic acids, the flavored soy sauce samples were diluted 4 times in distilled water and filtered through a 0.45 µm syringe filter. Free sugars were quantified by injecting 20 µL of the filtered solution into an HPLC system coupled with a refractive index detector (Alliance, Water Co.; Milford, MA, USA). The column was eluted with a calcium-ethylenediaminetetraacetic acid buffer solution (50 mg L^−1^) at a constant flow rate of 0.5 mL min^−1^. Identification and quantification of the different sugars were achieved by comparing the eluates against external sugar standards of known concentrations. The organic acids were quantified by injecting 20 µL of the flavored soy sauce sample into an HPLC system equipped with a refractive index detector (Shimadzu Co.; Kyoto, Japan), with 5 mM H_2_SO_4_ used as the mobile phase at a constant flow rate of 0.5 mL min^−1^.

For estimation of the free amino acids, the flavored soy sauce samples were first diluted 50 times in 0.2 N NaOH and then filtered through a 0.45-µm syringe filter. Finally, 20 µL of the sample was injected into an amino acid autoanalyzer (Hitachi; Tokyo, Japan).

### 2.7. Gas Chromatography-Mass Spectrometry (GC-MS) Analysis

The different compounds present in the various flavored soy sauce samples were extracted using the solid-phase microextraction technique and then analyzed by GC-MS, according to the method described by Lee et al. [17] with modifications. In brief, 5 mL of the flavored soy sauce sample in a closed vial was heated for 10 min at 70 °C to evaporate the volatile compounds, which were subsequently adsorbed onto a carbowax/divinylbenzene polydimethylsiloxane solid phase microextraction fiber assembly (SPME). GC was conducted on an Agilent 7890B GC–5977 GC/MSD system equipped with an Agilent DB-WAX 92-122-7062E column (60 m × 250 μm × 0.25 μm; Agilent; Santa Clara, CA, USA). The temperature of both the injection port and column was maintained at 250 °C. Each sample was injected at a spit ratio of 20:1, with a flow rate of 24 mL min^−1^ at 18.55 psi. The mass spectrometry readings were taken at 0.5-s scan intervals. The mass spectra of the compounds in the flavored soy sauce samples were compared with the spectra of known compounds stored in the National Institute of Standards and Technology MS library (2008 version).

### 2.8. Estimation of the Total Phenolics, Flavonoids, and In Vitro Antioxidative Activity

The total phenolics in the flavored soy sauce samples were determined using the Folin–Ciocalteu method [18], whereas the total flavonoids were quantified according to the method described by Lamien-Meda et al. [19]. The in vitro antioxidative potential of the samples was evaluated using 2,2-diphenyl-1-picrylhydrazyl (DPPH) and ferric-reducing antioxidant power (FRAP) assays [17].

### 2.9. Cellular Antioxidative Potential

#### 2.9.1. Cell Culture and Cell Viability Assay

Mouse fibroblast National Institute of Health (NIH) 3T3 cells were purchased from the American Type Culture Collection (Manassas, VA, USA) and maintained in Dulbecco’s modified Eagle’s medium (supplemented with 10% (*v/v*) fetal calf serum and a 1% penicillin-streptomycin cocktail) at 37 °C in a 5% CO_2_ incubator. The effects of different concentrations (0–50 µL mL^−1^) of the flavored soy sauce samples on the viability of the NIH 3T3 cells were evaluated using the 3-(4,5-dimethylthiazol-2-yl)-2,5-diphenyltetrazolium bromide (MTT) assay.

#### 2.9.2. Determination of Cellular Reactive Oxygen Species (ROS)

NIH 3T3 cells were grown in 24-well culture plates until 80% confluency and then treated with 1.5 µL mL^−1^ of the flavored soy sauce samples. After 24 h incubation, oxidative stress was induced in the cells by the administration of 0.25 mM H_2_O_2_ for 2 h. Finally, the cells were washed with 0.1 M phosphate-buffered saline (PBS, pH 7.4) and stained with 20 µM 2′,7′-dichlorofluorescein diacetate (DCFDA) for 30 min. After washing twice with 0.1 M PBS, the stained cells were visualized immediately under an epifluorescence microscope (Nikon; Kanagawa, Japan) for the production of reactive oxygen species. The fluorescence intensity was quantified using ImageJ software (National Institutes of Health; Bethesda, MA, USA).

### 2.10. Enumeration, Identification, and Enzyme Profiling of the Lactic Acid Bacteria

The presence of lactic acid bacteria in the flavored soy sauce samples was determined by spreading different dilutions on de Man, Rogosa, and Sharpe agar medium (Difco; Sparks, MD, USA). The isolated strains of lactic acid bacteria were Gram stained to determine their size and shape. The bacterial cells were also processed for molecular characterization through 16S rRNA gene sequencing (Solgent Co., Ltd.; Daejeon, Korea). The 16S rRNA gene sequences were compared with those available on the GenBank database using the Basic Local Alignment Search (BLAST) tool, and a phylogenetic tree was constructed with the neighbor-joining method using MEGA6.0 software [20]. The enzymes produced by the isolated lactic acid bacteria were evaluated using the API ZYM Kit (bioMerieux; Marcy-l’Étoile, France) according to the manufacturer’s instructions.

### 2.11. Xanthine Oxidase Inhibitory Activity of the Lactic Acid Bacteria

Tests on the xanthine oxidase inhibitory activity of the lactic acid bacteria were carried out using the cell-free culture supernatant and cell-free extract of the isolated bacteria. To prepare the cell-free culture supernatant, overnight-grown cultures of the lactic acid bacteria were centrifuged at 6000× *g* for 10 min, the supernatant was collected, and its pH was adjusted to 7.4 with 0.1 N NaOH. The supernatant was freeze-dried and finally suspended in 0.1 M phosphate buffer (pH 7.8). To prepare the cell-free extract, the bacterial pellet was washed twice with 0.1 M phosphate buffer (pH 7.8) and sonicated for 10 min on ice at 3–10 s pulses. Finally, the cell lysate was centrifuged at 8000× *g* for 10 min and processed for freeze drying.

Inhibition of the enzyme activity was evaluated by mixing various concentrations of cell-free culture supernatant or cell-free extract (0.25–2.5 mg mL^−1^) with 0.2 U mL^−1^ xanthine oxidase and 0.2 mM xanthine in 0.1 M phosphate buffer (pH 7.8). Finally, the absorbance was recorded at 290 nm. A control devoid of cell-free culture supernatant and cell-free extract was set up under the same experimental conditions. The percentage of xanthine oxidase inhibition was calculated with the following equation:

inhibition (%) = (1 − *A*_s_/*A*_c_) × 100 (1)

where *A*_s_ is the absorbance of the sample in the presence of cell-free culture supernatant or cell-free extract and *A*_c_ is the absorbance of the control.

### 2.12. Quantification of Biogenic Amines

Biogenic amines in different flavored soy sauce samples were examined by the method adopted by Shukla et al., 2015 [16]. In brief, 1 mL of flavored soy sauce samples was mixed with 5 mL of 0.4 M perchloric acid and filtered through Whatman paper no. 1. The 1 mL of the filtrate was mixed with 200 μL of 2 M NaOH, 300 μL of a saturated solution of NaHCO_3_, and 2 mL of 0.04 M of dansyl chloride and incubated at 40 °C for 45 min. Subsequently, 100 μL of 25% NH_4_OH was added, and the volume of the mixture was adjusted to 5 mL using acetonitrile. Finally, the mixture was centrifuged at 2500× *g* for 5 min. The obtained supernatant was filtered through a 0.2 µm syringe filter and processed for HPLC analysis (Dionex Ultimate 3000 UHPLC+ focused, Thermo Fisher Scientific; Waltham, MA, USA). The biogenic amines were separated over the C_18_ column (5 µm pore size, 4.6 mm internal diameter, 250 mm length) using 0.1 M ammonium acetate and acetonitrile mobile phase with gradient programming up to 35 min. The separated biogenic amine peaks were analyzed at 254 nm and quantified using the known standards.

### 2.13. Statistical Analysis

All the experiments were carried out in triplicate, and the results are expressed as the mean ± standard deviation of three independent experiments. Statistical significance was calculated using one-way analysis of variance, followed by Duncan’s post-hoc test, using SPSS16 software (SPSS Inc.; Chicago, IL, USA). A value of *p* < 0.05 was considered as indicating statistical significance.

## 3. Results and Discussion

### 3.1. Analysis of the Physicochemical Properties of the Flavored Soy Sauces Prepared at Laboratory Scale

Four different types of flavored soy sauce were prepared using varying proportions of radishes, apples, and pears (Table 1), and their physiochemical properties were analyzed. The pH is the most important factor in the soy sauce fermentation process for the commencement of several biochemical events. An acidic pH environment, ranging from 4.85 to 5.47, was recorded in all the flavored soy sauce samples after six months of fermentation (Table 2). In all the samples, the pH value decreased progressively during the first two months of fermentation, likely as a result of the formation of various organic acids during the initial stage of fermentation [21]. However, the pH value increased after two months, suggesting proteolysis and the release of ammonia [22]. The pH changes during the fermentation process suggest the occurrence of various biochemical changes.

In addition to the pH, the quantity of soluble solids is an important physicochemical characteristic of fermented foods. Soluble solids comprise inorganic and organic materials, which vary greatly depending on the fermentation conditions [10]. The highest amount of soluble solids (40.60 °Bx) was detected in the traditional Korean soy sauce (control), whereas the lowest amount was found in FSS-C (31.60 °Bx) after six months of fermentation (Table 2). A negative correlation (*y* = −0.517*x* + 45.7, *r*^2^ = 0.89) between the total fruit or vegetable weight added and soluble solids content was observed for all the flavored soy sauce samples.

The browning of soy products occurs through enzymatic and non-enzymatic processes, such as the Amadori rearrangement and the Maillard reaction [15,16]. All four flavored soy sauce products showed browning capacity, which increased with the progression of fermentation (Table 2). These findings are supported by a similar study that showed the browning potential of edible insect-supplemented soy sauce [15].

### 3.2. Sensory Evaluation

Sensory evaluation was carried out as a mandatory concern of foods toward its consumer acceptability, and the results are depicted in Figure 2. There are many parameters to judge the sensory quality, which varies greatly with the types of food [16]. In all four combinations of flavored soy sauce, FSS-D achieved the highest sensory score for flavor (5.08 ± 1.34), salty taste (5.16 ± 1.09), and sweet taste (4.88 ± 1.21), while the control traditional Korean soy accredited the least. Similar to this, a significantly better overall acceptability was observed for the FSS-D (5.81 ± 0.72) (*p* < 0.05). Sensory results collectively acclaimed FSS-D as the best preparation. We speculate that the different sensory properties of the tested flavored soy sauces were due to the differences in their basic composition and microbial diversity in it that generated different end products such as organic acids, sugars, and amino acids which are the well-known sensory components responsible for sensory quality. 

### 3.3. Comparative Study of Plant Scale Produced Flavored Soy Sauce with Commercial Flavored Soy Sauces

At the laboratory scale, FSS-D proved to have good taste and sensory qualities (Figure 2), while the physiochemical parameters were comparable with the traditional Korean soy sauce (control) and other three flavored soy sauce combinations (Table 2). Hence, FSS-D was selected as the best combination for the mass-level production at the plant scale (for methodology refer to Supplementary Materials S1.1) and compared with the two most popular commercial Korean flavored soy sauces, named as CFSS-A and CFSS-B.

### 3.4. Analysis of the Physicochemical Properties

**Figure 3:**
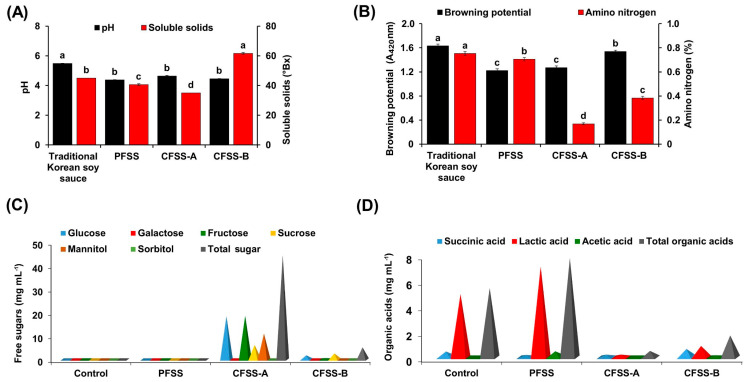
Comparison of the physicochemical properties of the final flavored soy sauce produced at plant scale (PFSS) with those of the control (traditional Korean soy sauce) and commercial flavored soy sauces (CFSS-A and CFSS-B). (**A**) pH and soluble solids. (**B**) Browning potential and amino nitrogen. (**C**) Free sugars. (**D**) Organic acids. Different lowercase letters on a bar indicate significantly differences (*p* < 0.05).

An acidic pH in the range of 4.38 ± 0.02 to 5.49 ± 0.01 was observed in all the samples after six months of fermentation (Figure 3A). The highest pH value was observed in the control soy sauce (5.49 ± 0.01) and the lowest in PFSS (4.38 ± 0.02). There was no significant difference in the pH among the PFSS (after six months of fermentation) and the two CFSS products. The soluble solids content varied significantly (*p* < 0.05) among the different soy sauces, with the highest amount measured in CFSS-B (61.66 ± 0.58 °Bx) and the lowest in CFSS-A (35.00 °Bx) (Figure 3A). The highest browning potential was observed in the control traditional Korean soy sauce (1.63 ± 0.03), whereas the lowest was seen in PFSS (1.22 ± 0.03) (Figure 3B).

The amino nitrogen content reflects the quality of soy sauce and is a common measure for assessing the progress of fermentation, particularly for protein-rich material. Moreover, it has a great impact on the flavor/aroma of the final product [23]. In China, the grading of soy sauce is based on the amino nitrogen content, where products with a content higher than 0.7 g 100 mL^−1^ are considered to be the best (grade A) [10]. Traditional Korean soy sauce (control) showed the highest amount of amino nitrogen content (0.75 ± 0.02%), followed by PFSS (0.71 ± 0.01%), signifying intense proteolytic and deaminase activity leading to the formation of small peptides, amino acids, and ammonia. In contrast, commercial flavored soy sauce samples, CFSS-B (0.38 ± 0.01%) and CFSS-A (0.17 ± 0.01%), showed a significantly low amount of amino nitrogen (*p* < 0.05) (Figure 3B).

### 3.5. Quantification of Total Sugars and Organic Acids

Aside from having nutritional value, free sugars are key components that determine the flavor and taste of fermented soy products [16]. We assayed the soy sauces for the presence of sucrose, glucose, galactose, fructose, mannitol, and sorbitol and observed different amounts in the samples (Figure 3C, Supplementary Figure S2). A markedly high amount of total free sugars was found in CFSS-A (44.38 mg mL^−1^), followed by CFSS-B (5.19 mg mL^−1^), and the lowest amount was in PFSS (0.05 mg mL^−1^). Among the different estimated sugars, glucose was present in all the samples with abundance in CFSS-A (18.51 mg mL^−1^), followed by CFSS-B (1.80 mg mL^−1^), traditional Korean soy sauce (control) (0.17 mg mL^−1^), and least in PFSS (0.05 mg mL^−1^) (Figure 3C). Galactose (0.13 mg mL^−1^) and sorbitol (0.02 mg mL^−1^) were present only in the control soy sauce and CFSS-B, respectively (Figure 3C). Fructose, sucrose, and mannitol were detected only in CFSS-A and CFSS-B (Figure 3C). A 31-fold higher fructose (18.62 mg/mL^−1^), 2.4-fold higher sucrose (6.18 mg mL^−1^), and 5.4-fold higher (1.07 mg mL^−1^) mannitol was quantified in CFSS-A in contrast to CFSS-B. We speculate that the difference in the sugar content in all the soy sauce samples was due to the involvement of different microbial enzymes or the difference in the raw materials used for the preparation of the product.

The diverse microbial population in the soy sauce acts on free amino acids and sugars, leading to the formation of various important organic acids that are not only responsible for the taste and aroma but also vital for many biochemical reactions and numerous health benefits [24,25]. Three different types of organic acids (namely, succinic acid, acetic acid, and lactic acid) were detected in the soy sauce samples (Figure 3D, Supplementary Figure S3). The highest amount of organic acids was observed in PFSS (7.77 mg mL^−1^), followed by the control (5.43 mg mL^−1^), CFSS-B (1.71 mg mL^−1^), and CFSS-A (0.50 mg mL^−1^) (Figure 3D). Results were allied well with the earlier performed free sugar results and exhibited a negative correlation between the amount of total free sugars and organic acids, suggesting the conversion of sugars to organic acids. Moreover, the role of the lactic acid bacterium *Tetragenococcus halophilus* in the production of various organic acids has been documented [21]. Furthermore, the higher amount of total organic acids in PFSS may be attributed to the presence of the added fruits and vegetables, which are considered as chief sources of organic acids or their precursor molecules [26].

Lactic acid was the most abundant of all the organic acids found, with the highest amount being in PFSS (7.11 mg mL^−1^), followed by the control (4.95 mg mL^−1^), CFSS-B (0.89 mg mL^−1^), and CFSS-A (0.21 mg mL^−1^) (Figure 3D). The most probable reason for the different lactic acid amounts among the different samples is the differences in the microbial populations (particularly lactic acid bacteria) that metabolize the simple sugars and convert them to organic acids and eventually effect the sensory and nutritional behavior of food. 

The presence of volatile acetic acid in soy products has been reported [27] and depends highly on the raw material used, pH, temperature, and microbial involvement [7]. Consistent with this, we also noticed the presence of acetic acid in all the soy sauce samples, with the highest amount being in PFSS (0.47 mg mL^−1^), followed by CFSS-B (0.16 mg mL^−1^), CFSS-A (0.08 mg mL^−1^), and the control (0.02 mg mL^−1^) (Figure 3D). Interestingly, PFSS had the lowest amount of succinic acid (0.19 mg mL^−1^), whereas CFSS-B had the highest (0.66 mg mL^−1^) (Figure 3D).

### 3.6. Quantification of Free Amino Acids

The amounts of total free amino acids in the different soy sauce samples are shown in Table 3. Other than the nutrient value, amino acids are also accountable for taste and color of the foods [28]. The highest amount of total free amino acids was observed in the control (183.93 mg mL^−1^), followed by PFSS (48.68 mg mL^−1^), and the lowest amount was observed in CFSS-A (24.18 mg mL^−1^). The difference in free amino acid profiles among the different soy sauces is likely due to the addition of different raw materials (substrates), which support the growth of different types of microbes with distinct proteases. The microbial action on the complex biomolecules, such as proteins, leads to the production of amino acids, which are one of the important constituents responsible for the sensory and nutritional quality of the foods [16]. We further characterized the free amino acids by dividing them into distinct groups on the basis of their taste (sweet, umami, bitter, and other) [16] and non-proteinogenic nature [29]. PFSS was found to contain a higher amount of sweet- and bitter-taste amino acids than CFSS-A and CFSS-B (Table 3). Furthermore, amino acids responsible for non-taste were also examined, and the highest amount was detected in control (65.97 mg mL^−1^), followed by CFSS-B (12.36 mg mL^−1^) and PFSS (10.16 mg mL^−1^), while the least was detected in the CFSS-A (6.34 mg mL^−1^). 

Amino acids belonging to non-proteinogenic types were also observed, whose essential biological role is well documented in the literature [29]. For example, *γ*-amino-*n*-butyric acid (GABA) is a well-known inhibitory neurotransmitter of the mammalian central nervous system [30] and a main dietary supplement [31]. We observed the highest amount of GABA in PFFS (7.71 mg mL^−1^) and the least in CFSS-B (0.10 mg mL^−1^). We speculate the higher amount of GABA in PFSS was due to its microbial profile, particularly the lactic acid bacteria, whose important role as a producer of GABA has been well documented [32,33]. The total non-proteinogenic amino acids were measured highest in PFSS (9.86 mg mL^−1^), followed by the control (6.19 mg mL^−1^), and the lowest amount being in CFSS-B (0.86 mg mL^−1^) (Table 3). The results collectively disclosed a good amount of non-proteinogenic and taste amino acids in PFSS, which could be effective in providing a specific taste to soy sauce along with the nutrient supplement.

### 3.7. GC-MS Profile of Flavored Soy Sauce

The results of the GC-MS analysis reveal the presence of variety of flavor compounds, primarily belonging to alcohol, esters, acids, acetaldehyde, ketones, phenolics, furan, pyrazine, as well as sulfur containing and other constituents, in flavored soy sauce (Supplementary Figures S4–S7). The retention times and amounts (% area) of the identified compounds are presented in Supplementary Tables S1–S4. The highest number of volatile compounds were identified in the control (127), followed by CFSS-A (117), PFSS (111), and CFSS-B (103). These differences might be attributed to the variation in raw materials and also the involvement of microorganisms that utilize those materials to produce different metabolites [21]. Among the different microbes, *T. halophilus* is known to contribute to the production of aromatic organic acids, alcohols, esters, and aldehydes in soy sauce [21]. In the control sauce, benzenemethanol (an aromatic alcohol) was the predominant compound (with a peak area of 12.03%), which is well known for its pleasant aroma and a precursor for corresponding ester and acid, followed by 2-undecanol (8.31%) (a fatty alcohol and flavoring agent) and 2-undecanone (7.58%) (a ketone class) (Table S1) and is responsible for a strong odor. By contrast, benzeneethanol was predominant in PFSS, with a peak area of 17.17%. Aside from its high prevalence in ensuring a pleasant aroma, benzeneethanol also serves as a precursor molecule for the formation of 2-phenethyl esters, which are natural flavoring agents. Additionally, PFSS also contained tetramethylpyrazine (2.16%), whose supportive roles in blood circulation, heart ailments, and headache relief as well as antioxidative activity have been well documented in the literature [34]. A high presence of 3-methyl-1-butanol (5.12%) (commonly known as isoamyl alcohol and an important aroma and sensory compound [21]) was detected in PFSS compared with the control. Another flavor/aroma compound, benzaldehyde, was observed in all the samples, with CFSS-B having the highest amount (9.94%). Benzaldehyde is considered a key volatile compound in soy products and is responsible for woody and nasty odors [35]. All four soy sauce samples also contained 3-methylbutanal, whose role as a key aroma component has been well documented in the literature [36]. The highest amount of 3-methylbutanal was detected in the control (3.22%), followed by PFSS (2.91%). Interestingly, ethanol was detected as the major flavor/aroma compound of CFSS-A, with a peak area of 17.36% (Table S3), whereas the other samples had much lower quantities of this compound. The importance of furanones as the major flavor/aroma constituents of soy sauce has been shown by Harada et al. [37]. Consistent with this, we also noticed different types of furanones in the tested samples. A high amount of 2(3H)-furanone, dihydro-5-(2-octenyl) (Z) (6.65%) was found in PFSS (Table S2). 

In summary, the GC-MS analysis revealed the diverse profiles of flavor and non-flavor volatile metabolites in the soy sauces that could further influence the taste, aroma, and nutritional and functional attributes of the product. The variations in the metabolite profiles of the different soy sauces might be due to the selection of different raw materials, which have an impact on the growth of microorganisms and fermentation. In particular, a correlation between *T. halophilus* and increases in certain flavor/aroma compounds during fermentation has been documented [21]. Our results concur with the findings of many reports that show the presence of similar compounds in soy sauce samples [6,10,38].

### 3.8. Total Phenolics, Flavonoids, and In Vitro Antioxidative Activity

**Figure 4:**
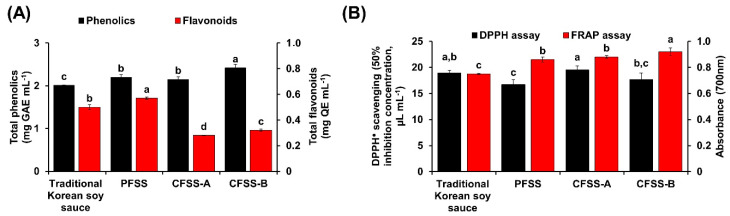
Comparison of the phenolics, flavonoids, and antioxidative activity of the final flavored soy sauce produced at plant scale (PFSS) with those of control (traditional Korean soy sauce) and commercial flavored soy sauces (CFSS-A and CFSS-B). (**A**) Total phenolics and flavonoids, expressed as gallic acid equivalents (GAE) mL^−1^ and quercetin equivalents (QE) mL^−1^ of soy sauce, respectively. (**B**) 2,2-Diphenyl-1-picrylhydrazyl (DPPH) and ferric reducing antioxidant power assays. The half maximal inhibitory concentration (IC_50_) value corresponds to the concentration achieving 50% DPPH radical-scavenging activity. Each value represents the mean ± standard deviation of three independent experiments. Values with different lowercase letters differ significantly from one another (*p* < 0.05).

Phenolics and flavonoids, which are secondary metabolites abundantly present in fruits and vegetables, have prominent health-promoting effects in humans. A high amount of phenolics were detected in CFSS-B (2.42 ± 0.08 mg gallic acid equivalents (GAE) mL^−1^), followed by PFSS (2.20 ± 0.06 mg GAE mL^−1^), whereas the lowest amount was in the control (2.01 ± 0.01 mg GAE mL^−1^) (Figure 4A). These results are in agreement with other published data, suggesting that the amount of phenolics in fermented soy products depends on the raw material chosen [39]. In addition to phenolics, a significantly high amount of flavonoids were detected in PFSS (0.57 ± 0.01 mg quercetin equivalents mL^−1^, *p* < 0.05) compared with that in the control and CFSS samples (Figure 4B).

Numerous diseases, such as cancer and rheumatoid arthritis, are directly associated with ROS-induced oxidative stress [40]. The clearance of ROS is usually carried out by many cellular enzymatic and non-enzymatic antioxidants. However, when a massive amount of ROS is generated, the resident antioxidants may prove to be insufficient for reducing the ROS to a basal level [40], and external antioxidants are required. Hence, the intake of antioxidant-rich foods would provide an excellent way to nullify the challenges posed by ROS. In this study, the antioxidative potential of the soy sauce samples was examined by DPPH assay. PFSS was revealed as a potent scavenger of DPPH radicals, with the lowest half maximal inhibitory concentration (IC_50_ = 16.70 ± 0.91 µL mL^−1^), which was 1.17 times lower (*p* < 0.05) than the IC_50_ value of CFSS-A (19.50 ± 0.80 µL mL^−1^) (Figure 4B). The better antioxidative activity of PFSS can be justified by the fact that it contains radishes, apples, and pears, which are rich sources of phytochemicals, whose potential role as antioxidants has been well documented in the literature [12,13]. Additionally, many reports suggest that the antioxidant activity of organic acids [41,42] may be a reason for the better antioxidant profiling of PFSS, due to high prevalence of organic acid in it. Meanwhile, the FRAP assay results suggest a strong electron-donating capacity of CFSS-B (0.92 ± 0.03), followed by CFSS-A (0.88 ± 0.01) and PFSS (0.86 ± 0.02) (Figure 4B). 

### 3.9. Cellular Antioxidative Potential

**Figure 5:**
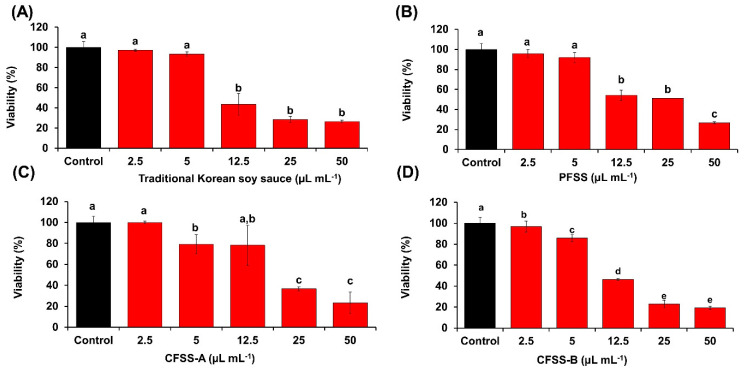
Effects of flavored soy sauce samples on the viability of NIH 3T3 fibroblasts, evaluated by 3-(4,5-dimethylthiazol-2-yl)-2,5-diphenyltetrazolium bromide (MTT) assay. The control represents cells without any soy sauce treatment. (**A**) Traditional Korean soy sauce. (**B**) Flavored soy sauce produced at plant scale (PFSS). (**C**,**D**) Commercial flavored soy sauces, CFSS-A and CFSS-B, respectively. Each value represents the mean ± standard deviation of three independent experiments. Values with different lowercase letters differ significantly from one another (*p* < 0.05).

**Figure 6:**
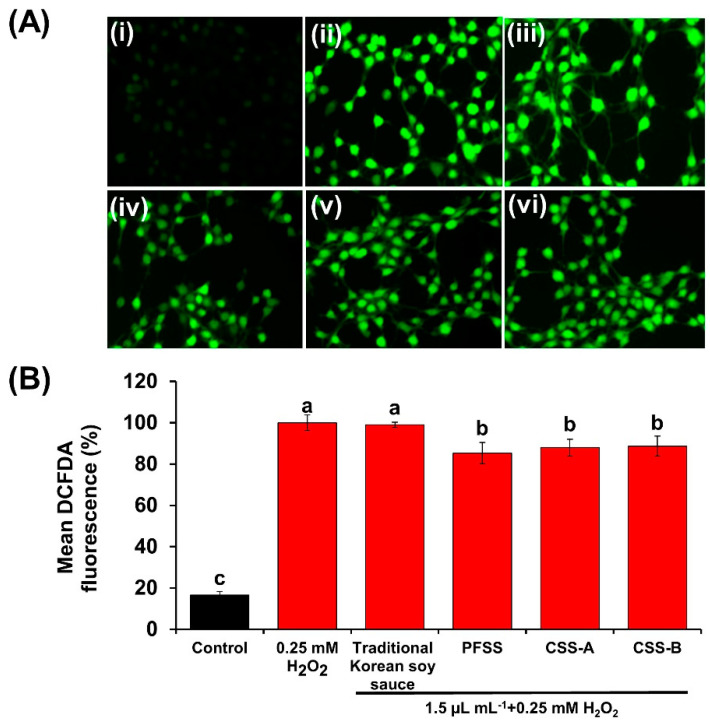
Effects of flavored soy sauce samples on the reactive oxygen species level in NIH 3T3 fibroblasts. (**A**) 2′,7′-Dichlorofluorescein diacetate (DCFDA) staining: (**i**) cells without H_2_O_2_ and soy sauce treatment (control), (**ii**) cells stimulated with 0.25 mM H_2_O_2_ only, (**iii**) cells treated with traditional Korean soy sauce + H_2_O_2_, (**iv**) cells treated with flavored soy sauce produced at plant scale (PFSS) + H_2_O_2_, (**v**) cells treated with commercial flavored soy sauce-A (CFSS-A) + H_2_O_2_, and (**vi**) cells treated with commercial flavored soy sauce-B (CFSS-B) + H_2_O_2_. (**B**) The fluorescence intensity quantified by Image J software. Each value represents the mean ± standard deviation of three independent experiments. Values with different lowercase letters differ significantly from one another (*p* < 0.05).

The effects of the flavored soy sauce samples on the viability of NIH 3T3 cells were determined by the MTT assay (Figure 5A–D). All the samples up to 2.5 µL mL^−1^ concentration had no significant adverse effect on the viability of the cells. Evaluation of the cellular antioxidative activity by DCFDA staining revealed that PFSS, CFSS-A, and CFSS-B were capable of inhibiting ROS, which was evident from the reduced fluorescent intensities (85.3 ± 5.1%, 87.9 ± 4.1%, and 88.6 ± 4.8%, respectively) relative to that of cells stimulated with H_2_O_2_ only (100%). By contrast, the control soy sauce did not show any significant cellular antioxidative activity (Figure 6A,B). These results suggest that PFSS is a better antioxidative product than the traditional Korean soy sauce and CFSS products. The most probable reason for this is the high amount of organic acids in PFSS and the presence of the fruits and vegetables, which are major sources of many important phytochemicals that are responsible for imparting numerous beneficial effects by directly scavenging ROS or inducing cellular antioxidants. There are a limited number of published studies on the cellular antioxidative potential of soy sauce. However, in one such study performed on a neuronal cell line, the antioxidative activity of bamboo salt-supplemented soy sauce in inhibiting ROS was demonstrated by DCFDA staining [43]. In a similar study by Jeong and Om [44], the protective role of different soy sauces against H_2_O_2_-induced oxidative stress was demonstrated in U373MG human astrocytes.

### 3.10. Enumeration, Identification, and Enzyme Profiling of Lactic Acid Bacteria

**Figure 7:**
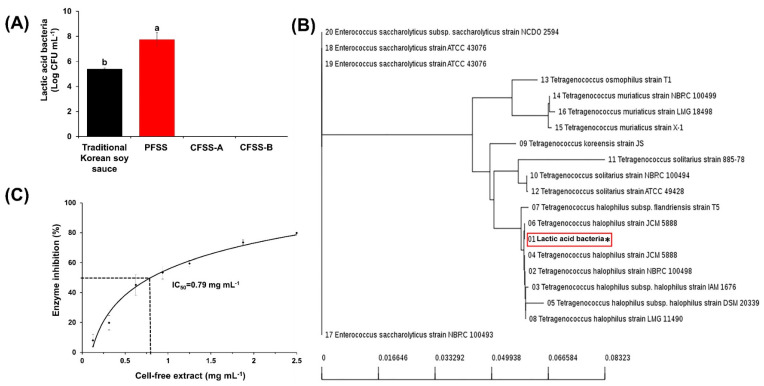
Enumeration, identification, and xanthine oxidase inhibitory potential of lactic acid bacteria. (**A**) Lactic acid bacterial populations in different soy sauce samples. Values with different lowercase letters differ significantly from one another (*p* < 0.05). (**B**) Phylogenetic tree of the isolated lactic acid bacteria. The tree was constructed by comparing the 16S rRNA gene sequence of isolated lactic acid bacteria from plant scale produced flavored soy sauce (PFSS) with those of the other strains present in database. (**C**) Xanthine oxidase inhibitory potential of the cell-free extract of the isolated lactic acid bacteria. The half maximal inhibitory concentration (IC_50_) was determined with a logarithmic equation (*y* = 25.05 ln(*x*) + 5.69). Each value represents the mean ± standard deviation of three independent experiments. * Isolated lactic acid bacteria from flavored soy sauce sample.

The roles of lactic acid bacteria in the fermentation of soy products are well documented in the literature, where they have a direct impact on the functional properties of food [45]. A significantly higher population of lactic acid bacteria were observed in PFSS (7.74 ± 0.55 log CFU mL^−1^) than in the control soy sauce (5.39 ± 0.08 log CFU mL^−1^). Surprisingly, no lactic acid bacteria were observed in CFSS-A and CFSS-B (Figure 7A). 

On the basis of the colony morphology, size, shape, and Gram staining, only a single type of lactic acid bacteria was observed in the PFSS and control samples. Using comparative phylogenetic analysis based on 16S rRNA gene sequencing, the lactic acid bacterium was further identified as the closest homolog of *T. halophilus* strain JCM 5888 (98% similarity) (Figure 7B). PFSS was supplemented with vegetables and fruits, such as apples, which also contain lactic acid bacteria [46]. However, it is unlikely that these native lactic acid bacteria appeared in the PFSS as soy sauce contains a high salt concentration (15–20%), which supports the idea that only the growth of halophilic lactic acid bacteria evolved during the fermentation. This corroborated the findings of a previous study that suggested a high prevalence of *T. halophilus* in soy sauce and other high-salted fermented products [47] and its involvement in the production of different organic acids and flavor/aroma compounds. The sequence of the isolated *T. halophilus* strain has been submitted to NCBI GenBank under the accession number MN270899. The high prevalence of *T. halophilus* in PFSS is correlated with our findings of a high amount of organic acids and diverse aroma compounds in this soy sauce product.

It is imperative that any microorganism used as a food additive must not produce any enzyme that is harmful to humans. Therefore, the comprehensive enzyme activity profile of the isolated *T. halophilus* was determined. This lactic acid bacterium produced beneficial enzymes, such as alkaline phosphatase, esterase, esterase lipase, and acid phosphatase (Table 4). Importantly, it showed no activity for α-chymotrypsin, β-glucuronidase, and *N*-acetyl- β-glucosaminidase, which are usually associated with intestinal ailments [48].

### 3.11. Xanthine Oxidase Inhibition

Xanthine oxidase is an enzyme that catalyzes the conversion of hypoxanthine to xanthine and further to uric acid and superoxide radicals. Thus, an excessive activity of xanthine oxidase is associated with many pathological conditions [49]. The cell-free extract of the isolated *T. halophilus* displayed xanthine oxidase inhibitory activity, with an IC_50_ value of 0.79 mg mL^−1^ (Figure 7C). However, no inhibition of this enzyme was shown by the cell-free culture supernatant of the isolated bacterium. Although the inhibitory potential of the *Lactobacillus* species against many enzymes has been well documented in the literature [48], to the best of our knowledge, this is the first report showing such potential of *T. halophilus* against xanthine oxidase.

### 3.12. Quantification of Biogenic Amines

The biogenic amines of the soy sauce samples are shown in Table 5. The highest amount of the total biogenic amines was detected in PFSS (980.85 ± 4.15 mg L^−1^) followed by the control (891.11 ± 12.17 mg mL^−1^), while the least was detected in CFSS-B (15.79 ± 0.81 mg mL^−1^). Individually, tryptamine, 2-phenylethylamine, putrescine, cadaverine, histamine, tyramine, spermidine, and spermidine were quantified in each sample (Table 5). The level of histamine in all the samples was in the range of 9.32 ± 0.54 to 468.65 ± 3.43 mg L^−1^ with a noteworthily high amount in control (468.65 ± 3.43 mg L^−1^) and PFSS samples (461.99 ± 0.99 mg L^−1^). Similar to histamine, putrescine was also detected in abundance in control and PFSS samples. The minimum level of histamine and putrescine was noticed in CFSS-B, which was approximately 2-fold lower compared to the amount detected in CFSS-A (Table 5). A diversified biogenic amine profile of the different samples was mainly due to the difference in raw material and subsequently the difference in microbial populations, which are the major producer of biogenic amines in fermented food [50,51].

## 4. Conclusions

On the basis of the above findings, we concluded that radishes, apples, and pears are suitable materials for influencing the soy sauce fermentation process and leading to the production of a flavored soy sauce with improved qualities. Our novel plant scale produced flavored soy sauce displayed a high content of flavonoids and a marked increase in antioxidants and lactic acid bacteria compared with the tested commercial flavored soy sauce products, suggesting that plant scale produced flavored soy sauce is more functionally active. Moreover, plant scale produced flavored soy sauce displayed a better profile of free amino acids, organic acids, and diverse volatile components due to its raw materials and distinct microflora and could be utilized for commercial production. Although there are few reports on the production of flavored soy sauce, to the best of our knowledge, this is the first comprehensive study demonstrating flavored soy sauce preparation by the addition of radish, apple, and pear and its comparative quality analysis.

## Figures and Tables

**Table 1:** List of flavored soy sauce prepared by mixing radish, apple, and pear in different proportions.

Flavored Soy Sauce (Sample Code)	Flavored Soy Sauce Was Prepared by Combining Various Traditional Korean Soy Sauce that Were Supplemented with Pulverized Radish (10%; *w/w*), Apple (30%; *w/w*), and Pear (30%; *w/w*) in Different Proportions
Control	Traditional Korean soy sauce (without any pulverized vegetable/fruit)
FSS-A	1:1:1 (Radish added soy sauce:pear added soy sauce:apple added soy sauce)
FSS-B	1:3:2 (Radish added soy sauce:pear added soy sauce:apple added soy sauce)
FSS-C	1:2:3 (Radish added soy sauce:pear added soy sauce:apple added soy sauce)
FSS-D	1:2:2 (Radish added soy sauce:pear added soy sauce:apple added soy sauce)

Traditional Korean soy sauce: 32% soybeans in 18% (*w/v*) salt solution.

**Table 2:** Physiochemical analysis of different flavored soy sauce during the fermentation at laboratory scale.

Physicochemical Parameter	Sample Code	Fermentation Time (Months)					
		0	1	2	4	5	6
pH	Control	5.37 ± 0.00 ^NS^	5.29 ± 0.01 ^NS^	5.39 ± 0.00 ^NS^	5.54 ± 0.00 ^NS^	5.37 ± 0.00 ^NS^	5.47 ± 0.00 ^NS^
	FSS-A	5.34 ± 0.02 ^NS^	5.10 ± 0.00 ^NS^	4.78 ± 0.00 ^NS^	4.94 ± 0.0 ^NS^	4.81 ± 0.01 ^NS^	4.94 ± 0.01 ^NS^
	FSS-B	5.34 ± 0.01 ^NS^	5.06 ± 0.00 ^NS^	4.65 ± 0.00 ^NS^	4.84 ± 0.01 ^NS^	4.71 ± 0.01 ^NS^	4.85 ± 0.00 ^NS^
	FSS-C	5.31 ± 0.02 ^NS^	5.09 ± 0.01 ^NS^	4.86 ± 0.0 ^NS^	5.20 ± 0.01 ^NS^	4.99 ± 0.00 ^NS^	5.12 ± 0.01 ^NS^
	FSS-D	5.35 ± 0.03 ^NS^	5.07 ± 0.00 ^NS^	4.95 ± 0.01 ^NS^	5.33 ± 0.00 ^NS^	5.14 ± 0.01 ^NS^	5.25 ± 0.00 ^NS^
Soluble solids (°Bx)	Control	38.07 ± 0.06 ^a^	38.33 ± 0.57 ^a^	38.77 ± 0.20 ^a^	39.40 ± 0.00 ^a^	39.83 ± 0.06 ^a^	40.60 ± 0.00 ^a^
	FSS-A	32.67 ± 0.06 ^b^	32.33 ± 0.57 ^b^	32.03 ± 0.06 ^b^	32.40 ± 0.00 ^b^	32.93 ± 0.06 ^b^	33.53 ± 0.15 ^b^
	FSS-B	32.53 ± 0.06 ^b^	31.00 ± 0.00 ^b^	30.73 ± 0.00 ^b^	30.63 ± 0.06 ^b^	31.43 ± 0.12 ^b^	31.83 ± 0.06 ^c^
	FSS-C	32.20 ± 0.10 ^b^	31.33 ± 0.58 ^b^	30.23 ± 0.06 ^b^	30.23 ± 0.06 ^b^	30.90 ± 0.10 ^b^	31.60 ± 0.00 ^c^
	FSS-D	31.40 ± 0.10 ^b^	32.67 ± 0.57 ^b^	31.83 ± 0.06 ^b^	31.90 ± 0.00 ^b^	32.27 ± 0.06 ^b^	32.63 ± 0.16 ^b,c^
Browning potential (A420 nm)	Control	1.58 ± 0.01 ^a^	1.35 ± 0.03 ^a^	1.37 ± 0.03 ^a^	1.82 ± 0.00 ^a^	2.03 ± 0.08 ^a^	2.63 ± 0.06 ^a^
	FSS-A	1.38 ± 0.01 ^b^	1.04 ± 0.03 ^b^	1.12 ± 0.02 ^b^	1.38 ± 0.00 ^b^	2.00 ± 0.03 ^a^	2.62 ± 0.05 ^a^
	FSS-B	1.35 ± 0.01 ^b^	1.05 ± 0.04 ^b^	0.99 ± 0.02 ^b^	1.17 ± 0.00 ^c^	1.64 ± 0.03 ^b^	2.14 ± 0.05 ^c^
	FSS-C	1.34 ± 0.01 ^b^	0.99 ± 0.03 ^b^	1.05 ± 0.03 ^b^	1.40 ± 0.10 ^b^	1.80 ± 0.03 ^a^	2.24 ± 0.04 ^b^
	FSS-D	1.37 ± 0.01 ^b^	0.97 ± 0.02 ^b^	1.08 ± 0.03 ^b^	1.30 ± 0.00 ^b^	1.82 ± 0.06 ^a^	2.11 ± 0.05 ^c^

Each value represents mean ± standard deviation of three independent experiments. Mean values followed by different superscripts (a–c) in a column are significantly different. (*p* < 0.05). ^NS^ represent non-significant changes between the groups.

**Table 3:** Quantitative analysis of free amino acids in the plant scale produced flavored soy sauce (PFSS), traditional Korean soy sauce (control), and commercial flavored soy sauces (CFSS-A and CFSS-B).

Amino Acid (mg mL^−1^)		Sample			
		Traditional Korean Soy Sauce (Control)	PFSS ^(a)^	CFSS-A ^(b)^	CFSS-B ^(c)^
Sweet taste	Threonine	2.28	0.31	1.04	1.17
	Serine	3.40	ND ^(d)^	1.49	2.33
	Glycine	2.49	2.69	0.84	1.81
	Alanine	6.80	8.38	1.58	2.06
	Total	15.51	11.38	4.95	7.37
Umami taste	Aspartic acid	1.33	0.83	3.55	1.53
	Glutamic acid	14.53	0.87	3.09	15.17
	Cystine	0.12	ND	ND	ND
	Total	15.98	1.70	6.64	16.70
Bitter taste	Methionine	1.32	0.87	0.33	0.66
	Isoleucine	4.45	3.46	1.52	1.26
	Leucine	6.92	5.42	2.32	3.07
	Lysine	67.58	5.83	1.25	1.17
	Total	80.27	15.58	5.42	6.16
Non-taste	Tyrosine	0.85	0.19	0.28	1.02
	Phenylalanine	4.53	2.97	1.60	2.21
	Valine	4.68	4.55	1.65	1.4
	Histidine	0.36	0.02	0.28	0.54
	Proline	54.88	2.41	1.40	5.34
	Arginine	0.68	0.01	1.14	1.82
	Total	65.97	10.16	6.34	12.36
Non-proteinogenic amino acids	Ornithine	2.61	1.51	0.13	0.25
	β-Amino isobutyric acid	0.36	0.23	0.10	0.32
	α-Amino adipic acid	0.88	0.32	0.06	0.13
	Citrulline	1.65	ND	0.23	ND
	γ-Amino-n-butyric acid	0.44	7.71	0.25	0.10
	Ethanol amine	0.16	0.06	0.03	0.03
	Hydroxylysine	0.09	0.04	0.02	0.03
	Total	6.19	9.86	0.82	0.86
	Grand total	83.93	48.68	24.18	43.46

^(a)^ PFSS: Plant scale produced flavored soy sauce, ^(b)^ CFSS-A: commercial flavored soy sauce A, ^(c)^ CFSS-B: commercial flavored soy sauce B. ^(d)^ ND: not detected.

**Table 4:** Enzymatic activity of the isolated *Tetragenococcus halophilus* (lactic acid bacterial strain) evaluated by the API ZYM kit.

Enzyme	Substrate	Enzyme Activity (nmol)
Alkaline phosphatase	2-naphthyl phosphate	>40
Esterase	2-naphthyl butyrate	20
Esterase lipase	2-naphthyl caprylate	20
Lipase	2-naphthyl myristate	0
Leucine arylamidase	L-leucyl-2-naphthylamide	0
Valine arylamidase	L-valyl-2-naphthylamide	0
Cystine arylamidase	L-cystyl-2-naphthylamide	0
Trypsin	N-benzoyl-DL-arginine-2-naphthylamide	0
α-Chymotrypsin	N-glutaryl-phenylanine-2-naphthylamide	0
Acid phosphatase	2-naphtyl phosphate	>40
Naphthol-AS-BI-phosphohydrolase	naphthol-AS-BI-phosphate	5
α-Galactosidase	6-Br-2-naphthyl-αD-galactopyranoside	0
β-Galactosidase	2-naphthyl-βD-galactopyranoside	0
β-Glucuronidase	naphthol-AS-BI-βD-glucuronide	0
α-Glucosidase	2-naphthyl-αD-glucopyranoside	0
β-Glucosidase	6-Br-2-naphthyl-βD-glucopyranoside	0
*N*-Acetyl-β-glucosaminidase	1-naphthyl-*N*-acetyl-βD-glucosaminide	0
α-Mannosidase	6-Br-2-naphthyl-αD-mannopyranoside	0
α-Fucosidase	2-naphthyl-αL-fucopyranoside	0

**Table 5:** Quantification of biogenic amines in traditional Korean soy sauce, plant scale produced flavored soy sauce (PFSS), and commercial flavored soy sauces (CFSS-A and CFSS-B) (mg L^−1^).

Biogenic Amine	Traditional Korean Soy Sauce (Control)	PFSS	CFSS-A	CFSS-B
Tryptamine	18.19 ± 0.00	13.92 ± 1.19	6.13 ± 0.07	0.96 ± 0.01
2-Phenylethylamine	20.69 ± 0.02	24.68 ± 0.77	2.16 ± 0.03	1.68 ± 0.02
Putrescine	348.28 ± 7.54	438.20 ± 0.42	3.74 ± 0.01	1.73 ± 0.03
Cadaverine	11.07 ± 0.46	13.97 ± 0.53	1.11 ± 0.02	0.57 ± 0.01
Histamine	468.65 ± 3.43	461.99 ± 0.99	18.68 ± 0.9	9.32 ± 0.54
Tyramine	23.49 ± 0.66	27.85 ± 0.16	3.10 ± 0.33	0.73 ± 0.02
Spermidine	0.67 ± 0.04	0.24 ± 0.09	2.35 ± 0.32	0.62 ± 0.13
Spermine	0.06 ± 0.02	0.00 ± 0.00	0.56 ± 0.06	0.18 ± 0.05
Total	891.11 ± 12.17 ^b^	980.85 ± 4.15 ^a^	37.27 ± 1.74 ^c^	15.79 ± 0.81 ^d^

Each value represents mean ± standard deviation of three independent experiments. Mean values followed by different superscripts in a column are significantly different (*p* < 0.05).

## Supplementary Materials

The following are available online at https://www.mdpi.com/2304-8158/9/11/1562/s1, Figure S1: Sensory evaluation of the soy sauce supplemented with radish, apple, and pear. Sensory evaluation was conducted by 15 participants and preference for the food was given on a scale of 1-5. Numeric value 1 represents the highest preference while numerical value 5 represents the least preference towards the food; Figure S2: Chromatogram of free sugars detected in various soy sauce samples. (A) Traditional Korean soy sauce (control). (B) Plant scale produced flavored soy sauce (PFSS). (C) Commercial flavored soy sauce-A (CFSS-A). (D) Commercial flavored soy sauce-B (CFSS-B); Figure S3: Chromatogram of organic acids detected in various soy sauce samples. (A) Traditional Korean soy sauce (control). (B) Plant scale produced flavored soy sauce (PFSS). (C) Commercial flavored soy sauce-A (CFSS-A). (D) Commercial flavored soy sauce-B (CFSS-B); Figures S4–S7: Gas chromatography-mass spectrometry chromatograms of traditional Korean soy sauce (control), Gas chromatography-mass spectrophotometry chromatogram of plant-scale produced flavored soy sauce (PFSS), Gas chromatography-mass spectrophotometry chromatogram of commercial flavored soy sauce (CFSS-A), and Gas chromatography-mass spectrophotometry chromatogram of commercial flavored soy sauce (CFSS-B); Tables S1–S4: Gas chromatography-mass spectrometry profiles of compounds detected in traditional Korean flavored soy sauce (control), Gas chromatography-mass spectrophotometry profile of compounds detected in the plant scale produced flavored soy sauce (PFSS), Gas chromatography-mass spectrophotometry profile of compounds detected in the commercial flavored soy sauce (CFSS-A), and Gas chromatography-mass spectrophotometry profile of compounds detected in the commercial flavored soy sauce (CFSS-B).

## References

[1] Ly D., Mayrhofer S., Domig K.J. (2018). Significance of traditional fermented foods in the lower Mekong subregion: A focus on lactic acid bacteria. Food Biosci..

[2] Shukla S., Park J., Park J.H., Kim M.K., Park S., Dubey A., Jeon J., Khang Y., Kim M. (2017). Evaluation of fungal microflora for aflatoxin producing possibility in novel quality *Meju* fermented with single and/or multiple additions of *Nelumbo nucifera, Ginkgo biloba*, and *Allium sativum* extracts. J. Food Saf..

[3] Kim H.G., Hong J.H., Song C.K., Shin H.W., Kim K.O. (2010). Sensory characteristics and consumer acceptability of fermented soybean paste (*Doenjang*). J. Food Sci..

[4] Yan Y.Z., Qian Y.L., Ji F.D., Chen J.Y., Han B.Z. (2013). Microbial composition during Chinese soy sauce koji-making based on culture dependent and independent methods. Food Microbiol..

[5] Kobayashi M. (2005). Immunological functions of soy sauce: Hypoallergenicity and antiallergenic activity of soy sauce. J. Biosci. Bioeng..

[6] Feng Y., Su G., Zhao H., Cai Y., Cui C., Sun-Waterhouse D., Zhao M. (2015). Characterization of aroma profiles of commercial soy sauce by odor activity value and omission test. Food Chem..

[7] Song Y.R., Jeong D.Y., Baik S.H. (2015). Effects of indigenous yeasts on physicochemical and microbial properties of Korean soy sauce prepared by low-salt fermentation. Food Microbiol..

[8] Shin D., Jeong D. (2015). Korean traditional fermented soybean products: Jang. J. Ethn. Foods.

[9] Choi U.K., Jeong Y.S., Kwon O.J., Park J.D., Kim Y.C. (2011). Comparative study of quality characteristics of Korean soy sauce made with soybeans germinated under dark and light conditions. Int. J. Mol. Sci..

[10] Xu D., Li C., Zhao M., Feng Y., Sun L., Wang Y. (2013). Assessment on the improvement of soy sauce fermentation by *Aspergillus oryzae* HG76. Biocatal. Agric. Biotechnol..

[11] Banihani S.A. (2017). Radish (*Raphanus sativus*) and diabetes. Nutrients.

[12] Boyer J., Liu R.H. (2004). Review: Apple phytochemicals and their health benefits. Nutr. J..

[13] Reiland H., Slavin J. (2015). Systematic review of pears and health. Nutr. Today.

[14] Stone H., Sidel J.L. (1985). Sensory Evaluation Practices.

[15] Cho J.H., Zhao H.L., Kim J.S., Kim S.H., Chung C.H. (2018). Characteristics of fermented seasoning sauces using *Tenebrio molitor* larvae. Innov. Food Sci. Emerg. Technol..

[16] Shukla S., Lee J.S., Park H.K., Kim J.K., Kim M. (2015). Effect of novel starter culture on reduction of biogenic amines, quality improvement, and sensory properties of *Doenjang*, a traditional Korean soybean fermented sauce variety. J. Food Sci..

[17] Lee J.S., Ramalingam S., Ji I.G., Kwon Y.S., Bahuguna A., Oh Y.S., Kwon O.-J., Kim M. (2018). Comparative study of the physicochemical, nutritional, and antioxidant properties of some commercial refined and non-centrifugal sugars. Food Res. Int..

[18] Singleton V.L., Orthofer R., Lamuela-Raventos R.M. (1999). Analysis of total phenols and other oxidation substrates and antioxidants by means of Folin-Ciocalteu reagent. Methods Enzymol..

[19] Lamien-Meda A., Lamien C.E., Compaoré M.M., Meda R.N., Kiendrebeogo M., Zeba B., Millogo J.F., Nacoulma O.G. (2008). Polyphenol content and antioxidant activity of fourteen wild edible fruits from Burkina Faso. Molecules.

[20] Tamura K., Stecher G., Peterson D., Filipski A., Kumar S. (2013). MEGA6: Molecular Evolutionary Genetics Analysis Version 6.0. Mol. Biol. Evol..

[21] Devanthi P.V.P., Gkatzionis K. (2019). Soy sauce fermentation: Microorganisms, aroma formation, and process modification. Int. Food Res. J..

[22] Battcock M., Azam-Ali S. (1998). Fermented fruits and vegetables: A global perspective. Food and Agriculture Organization Agricultural Services Bulletin.

[23] Ogueke C.C., Anosike F., Owuamanam C.I. (2015). Prediction of amino nitrogen during ugba (*Pentaclethra macrophylla*) production under different fermentation variables: A response surface approach. Niger. Food J..

[24] Dibner J.J., Buttin P. (2002). Use of organic acids as a model to study the impact of gut microflora on nutrition and metabolism. J. Appl. Poult. Res..

[25] Xu Y.T., Liu L.I., Long S.F., Pan L., Piao X.S. (2018). Effect of organic acids and essential oils on performance, intestinal health and digestive enzyme activities of weaned pigs. Anim. Feed Sci. Technol..

[26] Aprea E., Charles M., Endrizzi I., Corollaro M.L., Betta E., Biasioli F., Gasperi F. (2017). Sweet taste in apple: The role of sorbitol, individual sugars, organic acids and volatile compounds. Sci. Rep..

[27] Gao L., Liu T., An X., Zhang J., Ma X., Cui J. (2017). Analysis of volatile flavor compounds influencing Chinese-type soy sauces using GC-MS combined with HS-SPME and discrimination with electronic nose. J. Food Sci. Technol..

[28] Tseng Y.H., Lee Y.L., Li R.C., Mau J.L. (2005). Non-volatile flavor components of *Ganoderma tsugae*. Food Chem..

[29] Bahuguna A., Shukla S., Lee J.S., Bajpai V.K., Kim S.-Y., Huh Y.S., Han Y.-K., Kim M. (2019). Garlic augments the functional and nutritional behavior of *Doenjang*, a traditional Korean fermented soybean paste. Sci. Rep..

[30] Petroff O.A.C. (2002). GABA and glutamate in the human brain. Neuroscientist.

[31] Boonstra E., de Kleijn R., Colzato L.S., Alkemade A., Forstmann B.U., Nieuwenhuis S. (2015). Neurotransmitters as food supplements: The effects of GABA on brain and behavior. Front. Psychol..

[32] Cui Y., Miao K., Niyaphorn S., Qu X. (2020). Production of gamma-aminobutyric acid from lactic acid bacteria: A systematic review. Int. J. Mol. Sci..

[33] Ohmori T., Tahara M., Ohshima T. (2018). Mechanism of gamma-aminobutyric acid (GABA) production by a lactic acid bacterium in yogurt-sake. Process Biochem..

[34] Wu J., Zhao H., Du M., Song L., Xu X. (2019). Dispersive liquid-liquid microextraction for rapid and inexpensive determination of tetramethylpyrazine in vinegar. Food Chem..

[35] Peng X., Li X., Shi X., Guo S. (2014). Evaluation of the aroma quality of Chinese traditional soy paste during storage based on principal component analysis. Food Chem..

[36] Zhang H., Pu D., Sun B., Ren F., Zhang Y., Chen H. (2018). Characterization and comparison of key aroma compounds in raw and dry porcini mushroom (*Boletus edulis*) by aroma extract dilution analysis, quantitation and aroma recombination experiments. Food Chem..

[37] Harada R., Yuzuki M., Ito K., Shiga K., Bamba T., Fukusaki E. (2018). Microbe participation in aroma production during soy sauce fermentation. J. Biosci. Bioeng..

[38] Song Y.R., Jeong D.Y., Baik S.H. (2015). Monitoring of yeast communities and volatile flavor changes during traditional Korean soy sauce fermentation. J. Food. Sci..

[39] Shukla S., Park J., Park J.H., Lee J.S., Kim M. (2017). Development of novel *Meju* starter culture using plant extracts with reduced *Bacillus cereus* counts and enhanced functional properties. Sci. Rep..

[40] Valko M., Leibfritz D., Moncol J., Cronin M.T., Mazur M., Telser J. (2007). Free radicals and antioxidants in normal physiological functions and human disease. Int. J. Biochem. Cell Biol..

[41] Kayashima T., Katayama T. (2002). Oxalic acid is available as a natural antioxidant in some systems. Biochim. Biophys. Acta.

[42] Zhang B., Xia T., Duan W., Zhang Z., Li Y., Fang B., Xia M., Wang M. (2019). Effects of organic acids, amino acids and phenolic compounds on antioxidant characteristic of Zhenjiang aromatic vinegar. Molecules.

[43] Jeong J.H., Noh M.Y., Choi J.H., Lee H., Kim S.H. (2016). Neuroprotective and antioxidant activities of bamboo salt soy sauce against H_2_O_2_-induced oxidative stress in rat cortical neurons. Exp. Ther. Med..

[44] Jeong J.H., Om A.S. (2007). Specially-treated soy sauces regulate antioxidant activity and ROS in human astrocyte U373MG cells. Cancer Prev. Res..

[45] Abushelaibi A., Al-Mahadin S., El-Tarabily K., Shah N.P., Ayyash M. (2017). Characterization of potential probiotic lactic acid bacteria isolated from camel milk. LWT Food Sci. Technol..

[46] Savino M.J., Sánchez L.A., Saguir F.M., de Nadra M.C.M. (2012). Lactic acid bacteria isolated from apples are able to catabolise arginine. World J. Microbiol. Biotechnol..

[47] Jeong D.W., Heo S., Lee J.H. (2017). Safety assessment of *Tetragenococcus halophilus* isolates from doenjang, a Korean high-salt-fermented soybean paste. Food Microbiol..

[48] Son S.H., Jeon H.L., Yang S.J., Lee N.K., Paik H.D. (2017). In vitro characterization of *Lactobacillus brevis* KU15006, an isolate from kimchi, reveals anti-adhesion activity against foodborne pathogens and antidiabetic properties. Microb. Pathog..

[49] Muzychka O.V., Kobzar O.L., Popova A.V., Frasinyuk M.S., Vovk A.I. (2017). Carboxylated aurone derivatives as potent inhibitors of xanthine oxidase. Bioorg. Med. Chem..

[50] Naila A., Finlt S., Fletcher G., Bremer P., Meerdink G. (2010). Control of biogenic amines in food-existing and emerging approaches. J. Food Sci..

[51] Mah J.H., Park Y.K., Jin Y.H., Lee J.H., Hwang H.J. (2019). Bacterial production and control of biogenic amines in Asian fermented soybean foods. Foods.

