# Supplementary material for: Effects of Radishes, Apples, and Pears on the Lactic Acid Bacteria and Nutritional and Functional Qualities of Flavored Soy Sauce
Source: Foods. 2020 Oct 28;9(11):1562. doi: 10.3390/foods9111562 (PMC7694003; doi:10.3390/foods9111562)
Supplement: Supplementary file 1 [file foods-09-01562-s001.pdf]

Supplementary Materials

S 1.1. Preparation of flavored soy sauce

A) Flavored soy sauce preparation at laboratory scale

A traditional Korean soy sauce was prepared by using 32% soybeans *meju* in 18% salt solution and served as control and staring material for the preparation of flavored soy sauce. The radish, apple, and pears were washed and grinded by mechanical grinding and added separately in the basic ingredients of traditional Korean soy sauce (32% soybeans in 18% salt) at 10% (w/w), 30% (w/w), and 30% amount, respectively. The radish (10% radish+32% soybeans+18% salt), apple (30% apple+32% soybeans+18% salt), and pear (30% pear+32% soybeans+18% salt) supplemented soy sauces were further mixed in different proportions to make the four distinct flavored soy sauce. The 10% radish, 30% apple, and 30% pear supplemented soy sauces were mixed in equal amount (1:1:1) and designated as FSS-A. Further, the 10% radish, 30% apple, and 30% pear supplemented soy sauces were mixed in 1:3:2, 1:2:3, and 1:2:2 to make the three different flavored soy sauce and named as FSS-B, FSS-C, and FSS-D. Finally, all the formed FFS and traditional Korean soy sauce (control) preparations were fermented at ambient temperature for 6 months and filtered to separate solid portion to obtain liquid final product. For the laboratory scale production of flavored soy sauce was prepared at 600 mL.

B) Flavored soy sauce preparation at plant scale

The traditional Korean soy sauce at 100 L volume was prepared by supplementing 32% soybeans *meju* in 18% salt solution and served as control. The pulverized radish, apple, and pear were supplemented separately with the traditional Korean soy sauce at 10%, 30%, and 30% proportion (w/w), respectively. Radish (10%), pear (30%), and apple (30%)-supplemented soy sauces were finally mixed in 1: 2: 2 to make the flavored soy sauce at the plant scale (in 100 L volume) and fermented over the 6 months. This plant scale produced flavored soy sauce was named as PFSS.

S 1.2. Commercial flavored soy sauce composition

Two commercial flavored soy sauces, CFSS-A and CFSS-B, have the following compassions, respectively.

CFSS-A:

Skim soybean, wheat, sea salt, refined salt, starch syrup, fructose, mixed preparations (alcohol, emulsifier sodium lauryl sulfate, and vitamin B)

CFSS-B:

Skim soybeans, wheat, sea salt, starch syrup, green onion, garlic, ginger, fermented alcohol, pear concentrated fruit juice, apple concentrated fruit juice.

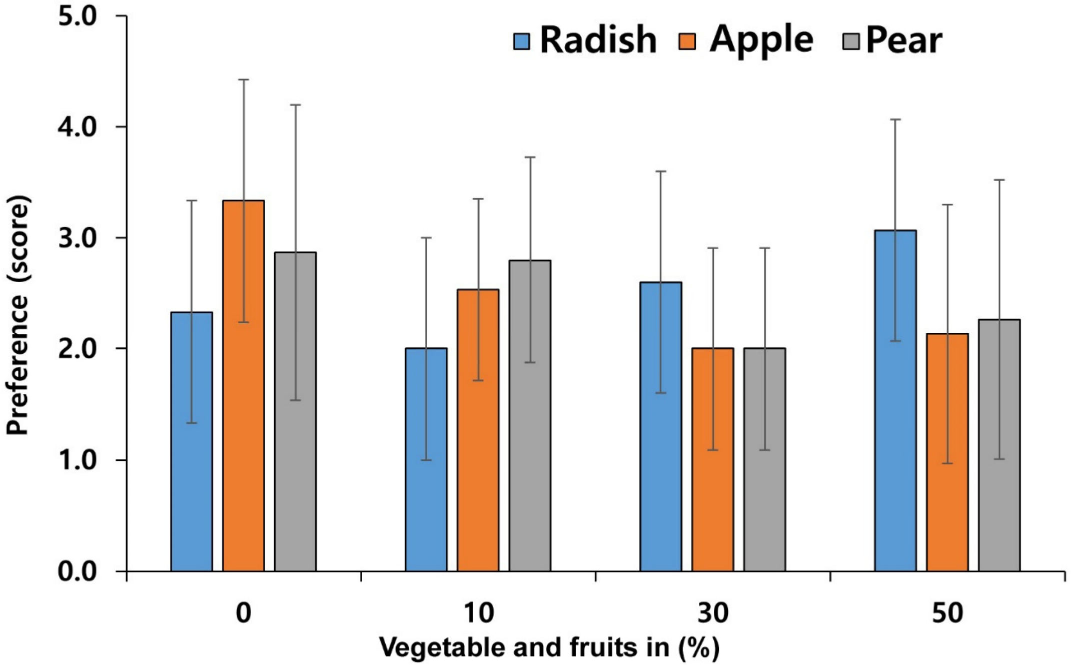

Figure 1. Sensory evaluation of the soy sauce supplemented with radish, apple, and pear. Sensory evaluation was conducted by 15 participants and preference for the food was given on a scale of 1-5. Numeric value 1 represents the highest preference while numerical value 5 represents the least preference towards the food.

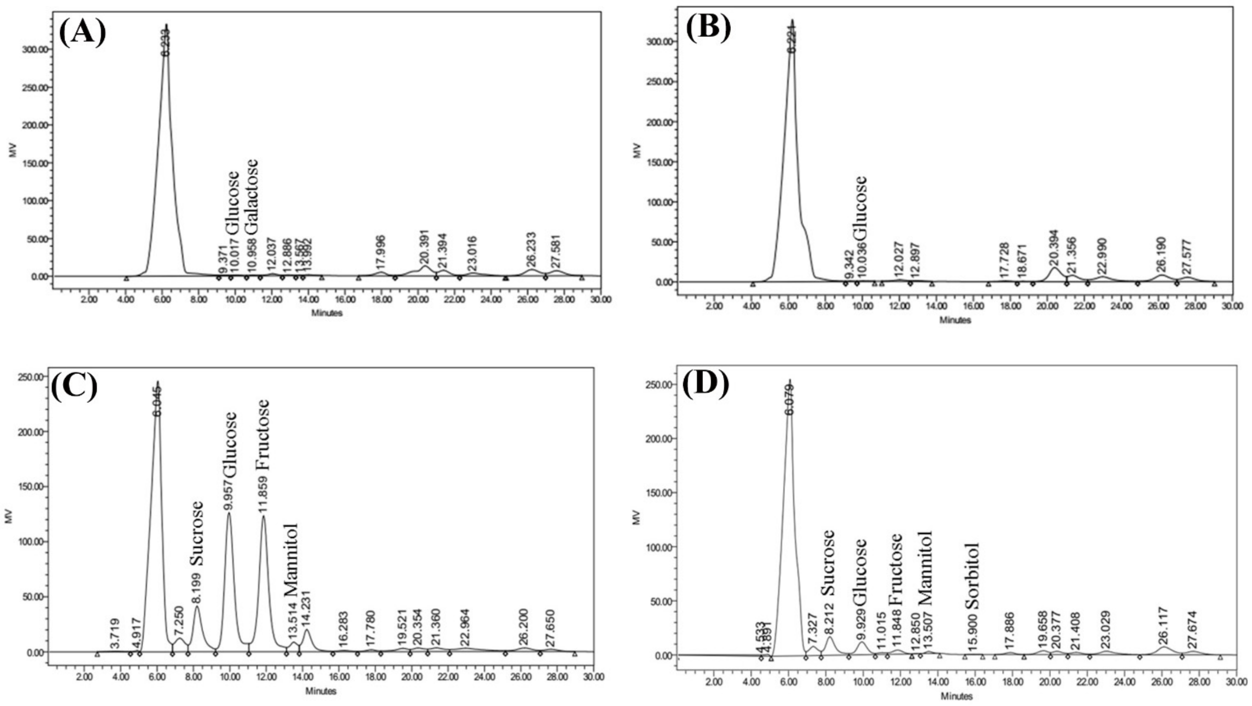

Figure 2. Chromatogram of free sugars detected in various soy sauce samples. **(A)** Traditional Korean soy sauce (control) **(B)** Plant scale produced flavored soy sauce (PFSS) **(C)** Commercial flavored soy sauce-A (CFSS-A) **(D)** Commercial flavored soy sauce-B (CFSS-B).

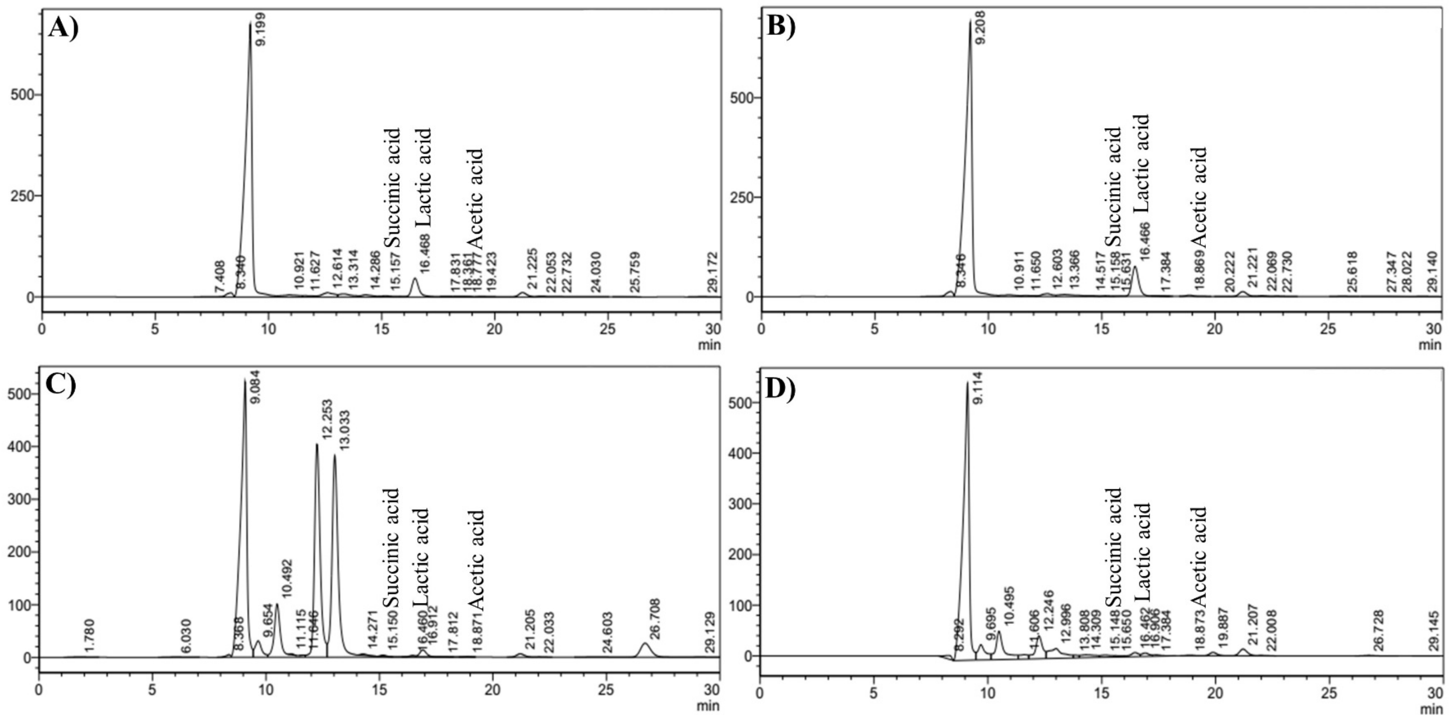

Figure 3. Chromatogram of organic acids detected in various soy sauce samples. **(A)** Traditional Korean soy sauce (control) **(B)** Plant scale produced flavored soy sauce (PFSS) **(C)** Commercial flavored soy sauce-A (CFSS-A) **(D)** Commercial flavored soy sauce-B (CFSS-B).

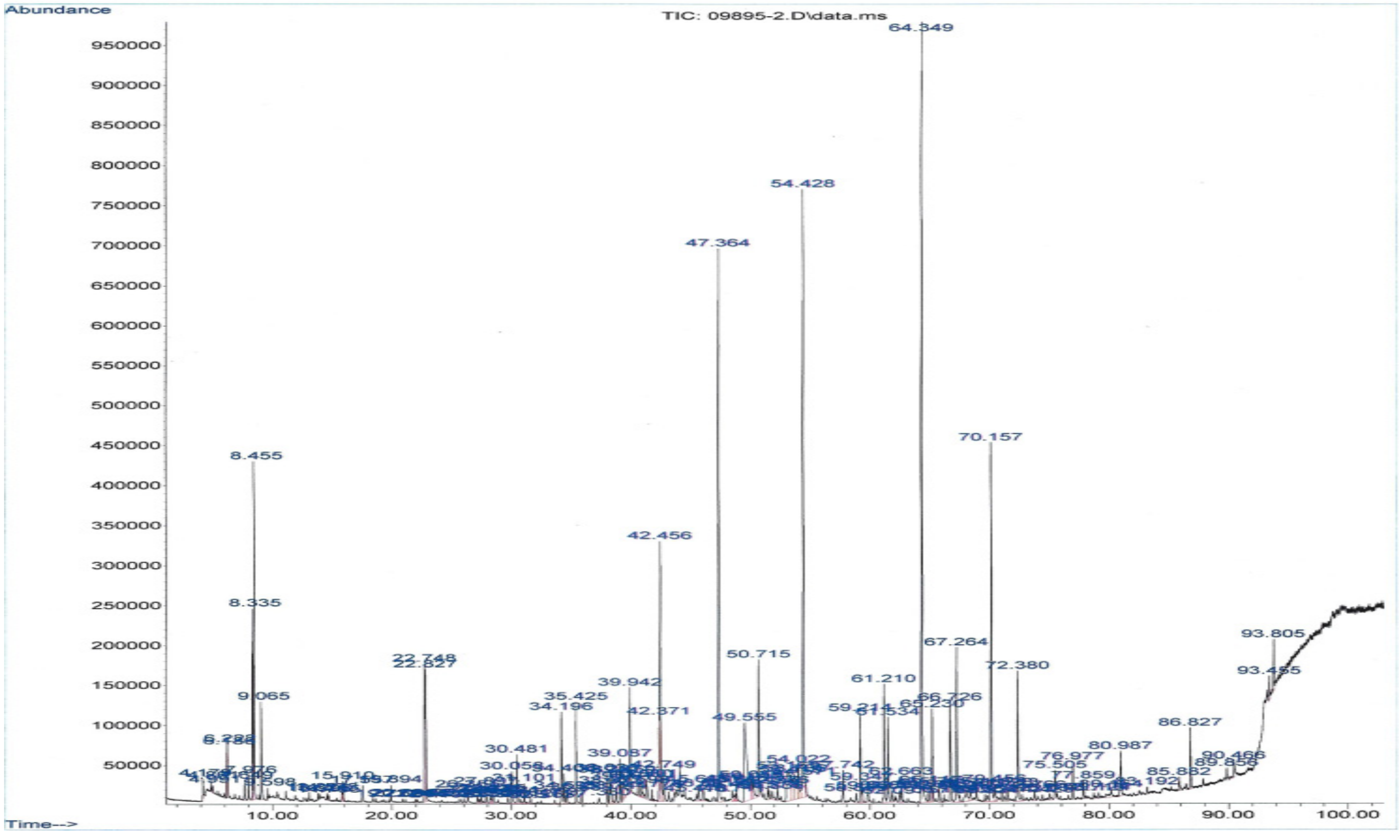

Figure 4. Gas chromatography-mass spectrophotometry chromatogram of traditional Korean soy sauce (control).

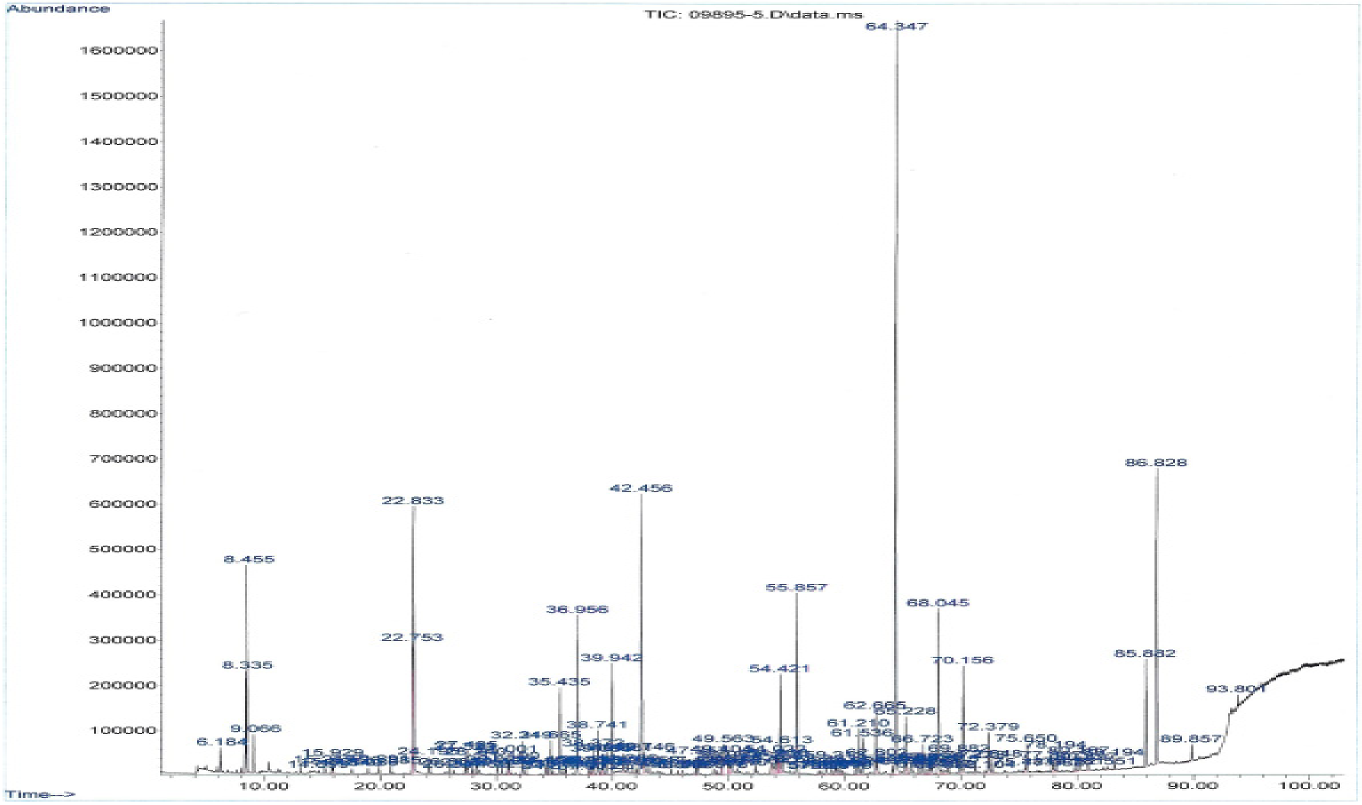

Figure 5. Gas chromatography-mass spectrophotometry chromatogram of plant scale prepared flavored soy sauce (PFSS).

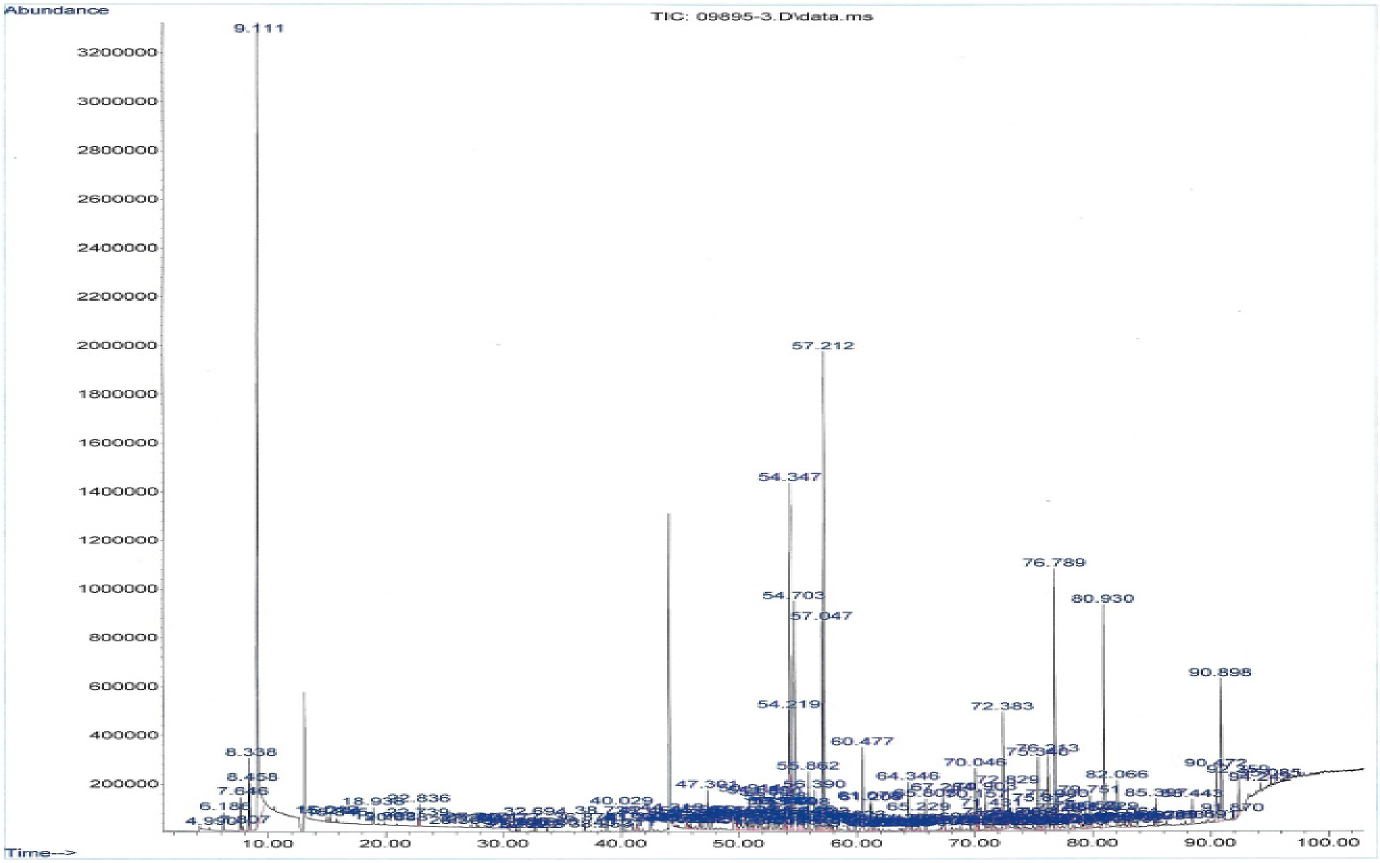

Figure 6. Gas chromatography-mass spectrophotometry chromatogram of commercial flavored soy sauce (CFSS-A).

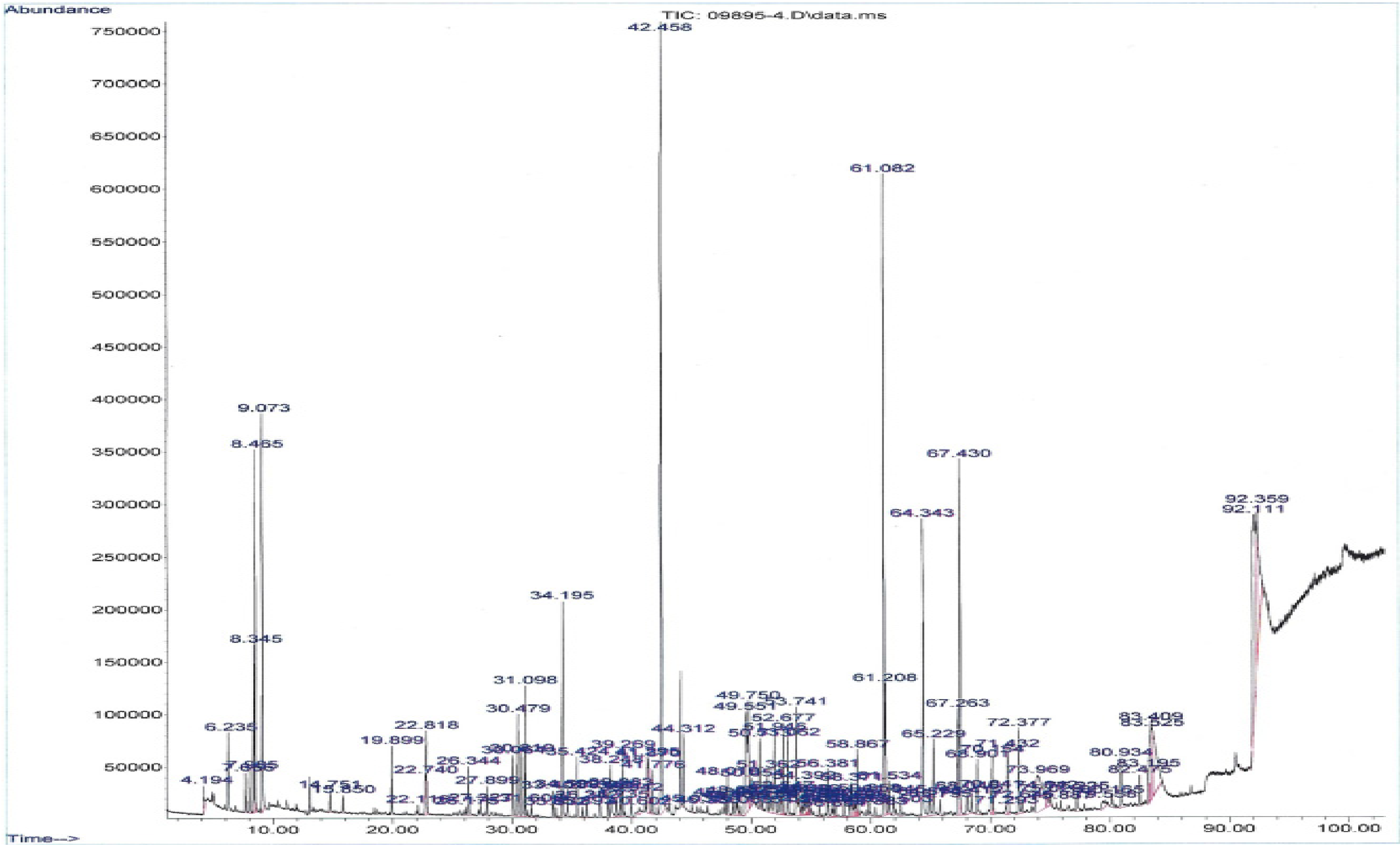

Figure 7. Gas chromatography-mass spectrophotometry chromatogram of commercial flavored soy sauce (CFSS-B).

Table 1. Gas chromatography-mass spectrophotometry profile of compounds detected in traditional Korean flavored soy sauce (control).

Compound number	Retention time	Percentage of area	Compound name
1	4.861	0.12	Methanethiol
2	4.991	0.10	Cyclobutanol
3	6.186	0.38	2-Methylpropanal
4	6.222	0.53	2-Propanone
5	7.649	0.24	Ethyl acetate
6	7.976	0.25	2-Butanone
7	8.335	1.74	2-Methylbutanal
8	8.455	3.22	3-Methylbutanal
9	9.065	1.24	Ethanol
10	9.598	0.08	2,5-Diethylfuraan
11	13.821	0.08	2-Methylbutanoic acid ethyl ester
12	14.573	0.07	Pentanoic acid, ethylester
13	14.705	0.08	Dimethyl disulfide
14	15.910	0.23	2-Methyl-1-propanol
15	17.557	0.26	3-Methyl-1-butanol acetate
16	19.722	0.02	1-Ethyl-1-cyclopentyloxy-1-silacyclopentane
17	19.894	0.16	Decamethylcyclopentasiloxane
18	21.098	0.04	Hyrdroxymethylapyrilene
19	22.118	0.05	Heptanal
20	22.748	1.71	2-Methyl-1-butanol
21	22.827	1.66	3-Methyl-1-butanol
22	25.670	0.09	Ethylbenzene
23	25.998	0.06	3-Methyl-2-hexanol
24	26.351	0.18	Methylpyrazine
25	27.163	0.11	2,3-Octanedione
26	27.330	0.06	Butanoic acid, 2-methyl-, 3-methylbutyl ester
27	24.475	0.10	*N*-(2-Hydroxyethyl)lactamide
28	27.897	0.22	Octanal
29	28.238	0.12	2H-Pyran, 3-4-dihydro
30	28.326	0.05	Ethylamine
31	28.485	0.19	Isopentyl 3-methylbutanoate
32	28.878	0.04	Methyl isobutyrate
33	28.878	0.11	4-Methyl-1-pentanol
34	30.058	0.57	2,5-Dimethylpyrazine
35	30.481	0.75	2,6-Dimethylpyrazine
36	30.812	0.07	Ethylpyrazine
37	31.101	0.22	Dodecamethylcyclohexasiloxane
38	31.617	0.08	2,3-Dimethylpyrazine
39	32.511	0.04	Sulfuric acid, diethyl ester-
40	33.637	0.06	Dimethyl trisulfide
41	34.196	1.32	2-Ethyl-6-methylpyrazine
42	34.406	0.40	2-Nonanone
43	34.595	0.23	2,4,6-Trimethylpyridine
44	35.425	1.40	Trimethylpyrazine
45	35.889	0.14	Dimethylphenylsilane
46	37.330	0.13	2,3-Dimethyl-5-ethylpyrazine
47	38.036	0.40	3-Ethyl-2,5-dimethylpyrazine
48	38.251	0.44	3-(Methylthio) propanal
49	38.373	0.29	Octen-3-ol
50	38.74	0.21	2-Furancarboxaldehyde
51	39.087	0.62	2,5-Dimethyl-3-ethylpyrazine
52	39.281	0.27	2-Methyl-6-propylpyrazine
53	39.459	0.68	2,3-Dimethyl-5-(1-methylethyl) pyrazine
54	39.942	1.48	Tetramethylpyrazine

55	40.880	0.23	2-Ethyl-1-hexanol
56	41.301	0.51	Decanal
57	41.778	0.11	Tetradecamethyl-cyclo-hepta-siloxane
58	42.371	0.76	2,3,5-Trimethyl-6-ethylpyrazine
59	42.456	4.47	Benzaldehyde
60	42.749	0.48	2-Nonanol
61	45.476	0.09	5-Methyl-2-furancarboxaldehyde
62	45.645	0.26	6-Methyl-[1,2,4]triazolo[4,3-b]pyridazine
63	46.061	0.25	2-Decanol
64	47.364	7.58	2-Undecanone
65	48.11	0.19	Methyl benzoate
66	47.711	0.09	Formic acid, 3-methylbut-2-yl ester
67	48.846	0.23	2-Isoamyl-6-methylpyrazine
68	49.555	4.03	Benzeneacetaldehyde
69	50.056	0.46	Phenyloxirane
70	50.218	0.48	Propylbenzene
71	50.715	1.98	2-Furanmethanol
72	51.456	0.18	2-Propyl-1-heptanol
73	51.578	0.10	Cyclododecasiloxane tertracosamethyl
74	51.836	0.14	2-Cyano-3,4-diethylpyrrole
75	52.26	0.20	2-Methylmerchaptomethylbut-2-enal
76	53.169	1.74	3-Methylbutanoic acid
77	53.419	1.14	Isovaleric acid
78	53.736	0.77	D-Allose
79	54.022	0.75	3-Methylthiopropanol
80	54.428	8.31	2-Undecanol
81	54.51	0.41	Diethylsilanediol
82	57.742	0.53	Ethyl 2-phenylacetate
83	58.855	0.18	6,7-Dihydro-2,3-dimethyl-5H-cyclopentapyrazine
84	59.214	1.21	2-Tridecanone
85	59.347	0.31	2-Phenylethyl acetate
86	59.860	0.23	4-Propylbenzaldehyde
87	61.210	1.82	3-Phenylfuran
88	61.534	1.36	2-Methoxyphenol
89	61.534	1.36	1-Cyclohexyl 1-4,5-dihydropyrazole
90	62.07	0.06	*N*-(3-Methylbutyl) acetamide
91	62.502	0.19	Benzyl alchol
92	62.663	0.34	4-Ethyl-1,2-dimethoxybenzene
93	64.349	12.03	Benzenemethanol
94	64.910	0.21	Benzeneacetonitrile
95	65.117	0.20	2-Heptadecanol
96	65.795	0.32	3-Methyl-1-naphthalenol
97	66.167	0.11	3-Phenyl-3,4,5,6-tetrahydro-2H-(1,2)-oxazine
98	67.264	2.50	1-(1H-Pyrrol-2-yl) ethanone
99	67.495	0.11	2,6-Pyridinedicarboxylicacid, isobutyl phenyl ester
100	68.047	0.41	2-Phenyl-1-butanol
101	68.283	0.23	2-O-(2-ethylhexyl) 1-O-(4-methylpentyl) oxalate
102	68.911	0.21	Phenol
103	69.634	0.10	Dehydromevalonic lactone
104	69.824	0.11	1H-Pyrrole-2-carboxyaldehyde
105	70.157	5.49	2-(2-methoxyphenyl) ethanol
106	70.456	0.36	4-Hydroxy-2,5-dimethyl-3(2*H*) furanone
107	71.028	0.21	3-Phenylpropanol
108	71.513	0.23	3-Methyl-2(1H)-quinoxalinone
109	72.380	1.95	5-Methyl-2-phenyl-2-hexenal
110	72.727	0.11	1,2,4-Trimethoxybenzene
111	73.766	0.18	3-Methylpyrrolo[1,2-a]pyrazine
112	74.425	0.09	1,6-Dimethyl-1H-indazole
113	75.031	0.13	2-Phenylthiophene

114	75.505	0.48	Juglone
115	76.977	0.59	*p*-Ethyl-phenol
116	77.859	0.27	2-Methoxy-4-vinylphenol
117	78.716	0.08	12-Crown-4
118	79.124	0.08	15-Crown-5
119	80.164	0.31	4-Phenylpyridine
120	80.987	0.84	2,6-Dimethoxyphenol
121	83.192	0.10	1,4,7,10,13,16,19-Heptaoxacyclohenicosan-2-one
122	85.882	0.22	Ethyltrimethylsilane
123	86.827	0.89	2(3H)-Furanone, dihydro-5-(2-octenyl), (Z)-
124	89.856	0.22	1,4,7,10,13,16-Hexacyclooctadecane
125	90.466	0.36	Octaethylene glycol monododecyl ether
126	93.455	0.29	3,6,9,12,15-Pentaoxanonadecan-1-ol
127	93.805	0.54	*N*-(2-Phenylethyl) acetamide

Table 2. Gas chromatography-mass spectrophotometry profile of compounds detected in the plant scale prepared flavored soy sauce (PFSS).

Compound number	Retention time	Percentage of area	Compound name
1	6.184	0.49	2-Methylpropanal
2	8.335	1.39	2-Methylbutanal
3	8.455	2.91	3-Methylbutanal
4	9.066	0.74	Ethanol
5	14.699	0.06	Dimethyl disulfide
6	15.215	0.16	Hexanal
7	15.827	0.12	2-Methyl-2-butenal
8	15.929	0.28	2-Methyl-1-propanol
9	17.549	0.15	1-Butanol, 3-methyl-, 1-acetate
10	18.930	0.10	2,5-Dimethyltetrahydrofuran
11	19.891	0.16	Decamethylcyclopentasiloxane
12	20.885	0.17	But-1-en-3-ynyl ethyl sulfide
13	22.753	2.21	2-Methyl-1-butanol
14	22.833	5.12	3-Methyl-1-butanol
15	24.195	0.30	2-Pentylfuran
16	26.001	0.14	• 2,3-Dihydrofuran
17	26.347	0.09	2-Methyl pyrazine
18	27.323	0.41	Isopentyl-2-methylbutanoate
19	27.485	0.48	2-Methylbutyl pentanoate
20	27.895	0.14	Octanal
21	28.240	0.33	1,4,5,6-Tetrahydropyridazine
22	28.452	0.12	Butanoic acid, 3-methyl-, 3-methylbutyl ester
23	30.074	0.25	2,5-Dimethylpyrazine
24	30.483	0.20	2,6-Dimethylpyrazine
25	31.001	0.39	6-Methyl-5-hepten-2-one
26	31.100	0.20	• Dodecamethylcyclohexasiloxane
27	32.219	0.70	1-Hexanol
28	34.196	0.20	2-Ethyl-6-methylpyrazine
29	34.405	0.18	2-Nonanone
30	34.655	0.72	Nonanal
31	34.877	0.07	3-OctanoI
32	35.435	1.94	4,6-Dimethyl-3-pyridinamine
33	35.890	0.10	2-Methyl-5-(1-methylethyl) pyrazine
34	36.005	0.13	3-Methylhexanol
35	36.956	3.23	2-Methylbutanoic acid, *n*-hexyl ester
36	37.917	0.06	2-(5-Methyl-5-vinyletrahydro-2-furanyl)-2-propanol
37	37.248	0.21	3-Methylthiopropanal
38	38.372	0.56	2,4,4-Trimethylpentanal
39	38741	1.13	2-Furan-carboxaldehyde

40	39.085	0.47	2-Ethyl-3,5-dimethylpyrazine
41	39.201	0.21	2-Methyl-6-hepten-1-ol
42	39.459	0.48	2-Amino-6-(*tert*-butyl) pyridine
43	39.942	2.16	Tetramethylpyrazine
44	40.251	0.06	Acetic acid
45	40.881	0.40	2-Ethylhexanol
46	41.289	0.27	Decanal
47	41.780	0.10	Tetradecamethyl cycloheptasiloxane
48	42.074	0.13	4-Methoxy-2,3,4-trimethyl-2-cyclobutanone
49	42.456	7.79	Benzaldehyde
50	42.746	0.47	2-Nonanol
51	45.029	0.13	Octyl formate
52	45.644	0.12	1,3-Dihydrobenoimidazol-2-one
53	46.053	0.17	3-(2-Pyridyl)-propanol
54	47.136	0.17	*p*-Fluorocarbanilonitrile
55	47.357	0.38	2-Undecanone
56	48.719	0.15	Hexyl tiglate
57	49.018	0.09	4,9-Decadienoic acid, 2-nitrohyl ester
58	49.563	2.29	Benzeneacetaldehyde
59	50.057	0.38	1-Phenyl-ethanone
60	5.214	0.37	Dimethylsilanediol
61	50.710	0.14	2-Furanmethanol
62	52.278	0.14	2-Methylmercaptomethylbut-2-enal
63	53.647	0.22	*β*-Alanine, N-methoxy, methyl ester
64	54.022	0.56	3-Methylpentanoic acid
65	54.238	0.56	3-Methylthiopropanol
66	54.238	0.36	1-Phenyl-1-propanon
67	54.310	0.25	1,2-Dimethoxybenzene
68	54.421	2.07	2-Undecanol
69	54.613	0.61	1-Methylene-4-(1-methylethenyl) cyclohexane,
70	57.738	0.09	Ethyl 2-phenylacetate
71	57.927	0.08	2-Methylpropyl pentyl sulfite
72	58.373	0.30	1,3,5,7-Cyclooctatetraene-1-carboxaldehyde
73	58.898	0.17	1-Phenyl-1,2-propanedione
74	59.213	0.32	2-Tridecanone
75	59.349	0.17	2-Phenylethyl acetate
76	60.924	0.19	2,7-Octadiene-1,6-diol, 2,6-dimethyl-
77	61.210	1.03	3-Phenylfuran
78	62.665	1.25	4-Ethyl-1,2-dimethoxybenzene
79	62.802	0.35	2,2,4-Trimethyl-1,3-pentanediol diisobutyrate
80	64.126	0.20	Acetic acid 4-acetoxy-6-methoxy-2-methyl-tetrahydro-pyran-3-yl ester
81	64.347	17.17	Benzeneethanol
82	65.228	1.47	2-Phenylbutanal
83	65.370	0.14	• 2,3-Dimethyl-3-hexanol
84	65.782	0.14	1-Naphtha1enemethano1
85	66.384	0.21	1-Phenyl-1-hexanone
86	66.723	0.78	2,6-Dimethyl-3-hdroxy-4H-pran-4-one
87	67.146	0.12	Oxan-3-ylacetic acid
88	67.264	0.28	1-(1H-pyrrol-2-yl) ethanone
89	67.498	0.34	2-Phenylethyl 2-phenylacetate
90	68.045	3.77	2-Phenylbutan-1-ol
91	68.367	0.19	2-Bromopropionic acid, 2-phenylethyl ester
92	68.696	0.20	2-Methyl-3-phenyl-2-propenal
93	68.915	0.24	Phenol
94	69.882	0.47	Tetrahydro-2-thiopheneacetonitrile
95	70.156	2.43	4-Ethylguaiacol
96	71.228	0.31	3-tert-Butyl-1,2-dihydronaphthalene
97	72.104	0.06	(2-Methoxyethenyl) benzene
98	72.379	0.93	5-Methyl-2-phenyl-2-hexenal
99	72.648	0.40	Indian-2-yl-methanol
100	75.481	0.17	1,2,3,4-Tetramethoxybenzene

101	75.650	0.63	γ-Decalactone
102	77.857	0.28	2-Methoxy-4-vinylphenol
103	77.982	0.09	5-Methyl-1,3-thiazole
104	78.194	0.47	6-Phenyldodecane
105	79.781	0.11	3-(2-Methylphenyl) cyclohexene
106	80.167	0.41	3-Phenylpyridine
107	80.897	0.35	Methyl heptanoate
108	82.551	0.12	1,4,7,10,13,16-Hexaoxacyclooctadecane
109	83.194	0.24	2,4-Di-*tert*-butylphenol
110	85.882	2.38	4-Octylbutan-4-olide
111	93.801	0.19	1,4,7,10,13,16-Hexathiacyclooctadecane

Table 3. Gas chromatography-mass spectrophotometry profile of compounds detected in the commercial flavored soy sauce (CFSS-A).

Compound number	Retention time	Percentage of area	Compound name
1	4.990	0.03	2-Propanamine
2	6.186	0.22	2-Methylpropanal
3	7.646	0.39	Ethyl acetate
4	7.807	0.06	1,1-Diethoxyethane
5	8.338	0.83	2-Methylbutanal
6	8.458	0.58	3-Methylbutanal
7	9.111	17.36	Ethanol
8	15.244	0.08	Methoxymethyl isothiocyanate
9	15.845	0.07	2,2-Dihydroxypropanedioic acid
10	15.938	0.26	3-Prop-2-enylsulfanylprop-1-ene
11	22.836	0.43	Eucalyptol
12	27.717	0.09	3-Cyclohexene-1-carboxylicacid
13	27.889	0.05	3-Methyl-2-heptanol
14	30.477	0.11	2,5-Dimethylpyrazine
15	31.000	0.03	6-Methyl-5-heptene-2-one
16	31.100	0.05	Dodecamethylcyclohexasiloxane
17	21.320	0.06	Ethyl 2-hydroxypropanoate
18	32.222	0.02	1-Hexanol
19	32.694	0.22	• 2-[2-(Methoxy)ethyl]-4(5)-methylimidazole
20	36.876	0.15	1-Allyl-2-isopropyldisulfane
21	38.253	0.03	3-(Methylthio) propanal
22	38.738	0.30	2-Furan-carboxaldehyde
23	40.029	0.43	3-(Prop-2-enyldisulfanyl) prop-1-ene
24	40.311	0.05	1,2,4-Metheno-1H-indene
25	40.999	0.11	Copaene
26	41.290	0.10	Decanal
27	42.459	0.33	Benzaldehyde
28	44.310	0.09	L-Linalool
29	44.690	0.05	Dimethylamine-D1
30	44.826	0.05	2-Hydroxyethyl propanoate
31	45.489	0.08	5-Methyl-2-furancarboxaldehyde
32	45.896	0.05	Benzoyl bromide
33	46.855	0.23	β-Elemene
34	47.301	0.73	Caryophyllene
35	47.614	0.29	1,3-Dithiolane
36	49.543	0.41	Benzeneacetaldehyde
37	49.667	0.06	Phenyloxirane
38	49.750	0.33	Ethyl decanoate
39	50.495	0.44	γ-Curcumene
40	50.713	0.19	2-Furanmethanol
41	51.402	0.36	1-(Propyltrisulfanyl) propane
42	51.689	0.63	Isoledene
43	52.389	0.15	Thujopsene-(I2)
44	52.650	0.82	1-Methyl-4-(-methylhept-5-en-2-yl)cyclohexa-1,3-diene
45	53.058	0.61	2-(4-Methyl-3-cyclohexen-1-yl)-2-propanol

46	53.340	0.44	1,7,7-Trimethylbicyclo[2.2.1]heptan-2-ol
47	53.744	0.44	Dodecanal
48	54.026	0.05	3-Methylthiopropanol
49	54.347	6.09	2-Methyl-5-(6-methylhept-5-en-2-yl) cyclohexa-1,3-diene
50	54.703	5.21	*β*-Bisabolene
51	55.008	0.64	3-Methyl-1,2,4-trithiolane
52	55.862	0.97	*α*-Farnesene
53	56.390	0.84	*δ*-Cadinene
54	56.723	0.25	(-)-*α*-Panasinse
55	56.896	0.21	Citronellol
56	57.047	3.49	*β*-Sesquiphellandrene
57	57.212	8.16	Benzene, 1-(1,5-dimethyl-4-hexenyl)-4-methyl-
58	57.594	0.15	Silana-3,7(11)-diene
59	57.746	0.18	Ethyl 2-phenylacetate
60	59.246	0.14	Ethanone, 1-(2-furanyl)-
61	59.402	0.04	Ethyl nicotinate
62	59.823	0.23	Benzenepropanoic acid, 2-ethenyl-4-methoxy-, methyl ester
63	61.078	0.54	Ethyl decanoate
64	61.209	0.51	Geraniol
65	61.540	0.08	2,5-Dimethyl-3-ethylfuran
66	61.836	0.04	3,5,11-Eudesmatriene
67	62.198	0.05	• Isothiocyanatoethane
68	62.742	0.04	*α*-Bromomesitylen
69	63.735	0.03	Dibutyl 3,6,9,12,15,18,21-heptaoxa tricosane-1,23-dioate
70	63.866	0.05	3,3-Diethoxy-1-propanol
71	64.346	0.93	Benzeneethanol
72	64.698	0.12	*α*-Calacorene
73	62.084	0.05	2,4-Dimethyl-2,4-pentadien-1-ol
74	65.229	0.36	2-Phenylbutanal
75	67.100	0.07	5-Methoxymethyl-[1,3,4]thiadiazol-2-ylamine
76	67.264	0.69	Ethanone, 1-(1H-pyrrol-2-yl)-
77	67.412	0.11	1-Pentamethyldisilyloxycyclopentan
78	67.984	0.09	Glutaric acid, dodec-2-en-1-yl 3-hexyl ester
79	98.451	0.08	Diglycolic acid, 2-chloro-6-fluorophenyl propyl ester
80	68.799	0.10	Trans-*β*-ionone
81	70.046	1.10	8-Amino-6-methoxy-2-methylquinolin
82	70.457	0.53	4-Ethylguaiacol
83	70.298	0.11	4-Aminopyridine-2-carboxylic acid
84	70.903	0.65	1,6,10-Dodecatrien-3-ol,3,7,11-trimethyl-
85	71.139	0.17	2-(2-Chloroethyl)-*m*-dithiane
86	72.207	0.04	Di-epi-1,10-cubenol
87	72.383	2.02	5-Methyl-2-phenyl-2-hexenal
88	72.602	0.20	3,5-Diethyl-1,2,4-trithiolane
89	72.829	0.74	Cyclohexanemethanol, 4-ethenyl-*α*,*α*,4-trimethyl-3-(1-methylethenyl)-, [1*R*-(1*α*,3*α*,4*β*)]-
90	73.356	0.17	Octaethylene glycol monododecyl ether
91	74.438	0.04	TMS derivative of hemiacetal oligomer of formaldehyde and methanol
92	74.912	0.09	2H-Thiopyran, 3,4-dihydro-2-[(2-propenylthio)methyl]-
93	75.044	0.06	1-Methylisophosphinoline
94	75.340	1.27	(+)-Calarene or (+)-β-Gurjune
95	76.459	0.36	Mintsulfide
96	76.789	4.56	6-Ethyl-4,5,7-trithia-2,8-decadien
97	77.836	0.49	(3-tert-Butylphenyl) carbamate
98	78.463	0.02	12-Crown-4
99	79.065	0.08	*β*-Himachalene
100	79.188	0.09	*α*-Bisabolol
101	79.848	0.32	*α*-Cadinol
102	80.660	0.09	1,4,7,10,13,16-Hexaoxacyclooctadecane

103	80.930	4.21	Ethyl hexadecanoate
104	81.229	0.42	3,4-Pyridinediamine
105	82.066	0.86	Ethyl 9-hexadecenoate
106	82.542	0.14	(E1)-1-(6,10-Dimethylundeca-5,9-dien-2-yl)-4-methylbenzebne
107	83.061	0.23	Methoxyacetic acid, TMS derivative
108	83.198	0.07	Phenol, 2,4-bis (1,1-dimethyl)
109	85.198	0.09	2-(bromomethyl)-2-hexyl-15-crown-5
110	85.397	0.51	Farnesol 1
111	86.248	0.09	1,4,7,10,13-Pentaoxacyclopentadecane
112	88.443	0.53	Ethanedioic acid, bis (trimethylsilyl) ester
113	90.472	1.21	Ethyl oleate
114	90.898	2.78	1-Allyl-3-(2-(allylthio)propyl) tri sulfane
115	91.870	0.12	21-Krone-7
116	92.359	0.64	Linoleic acid ethyl ester
117	97.406	0.13	15-Crown-5

Table 4. Gas chromatography-mass spectrophotometry profile of compounds detected in the commercial flavored soy sauce (CFSS-B).

Compound number	Retention time	Percentage of area	Compound name
1	4.194	0.15	Acetonitrile-d3
2	6.235	0.83	2-Propanone
3	7.656	0.32	Ethyl acetate
4	7.985	0.35	2-Butanone
5	8.345	1.18	2-Methylbutanal
6	8.465	2.69	3-Methylbutanal
7	9.073	3.58	Ethanol
8	14.751	0.23	Dimethyl disulfide
9	15.850	0.19	2-Methyl-1-propanol
10	19.899	0.49	Decamethylcyclopentasiloxane
11	22.116	0.09	Trimethyloxazole
12	22.740	0.36	2-Methyl-1-butanol
13	22.818	0.85	3-Methyl-1-butanol
14	26.175	0.09	2-Ethyl-3,4-dimethyl oxazole
15	26.34	0.59	2-Methyl pyrazine
16	27.327	0.11	4-Methylthiazole
17	27.899	0.29	Octanal
18	30.064	0.76	2,5-Dimethylpyrazine
19	30.479	1.14	2,6-Dimethylpyrazine
20	30.810	0.72	Ethylpyrazine
21	31.098	0.96	Dodecamethylcyclohexasiloxane
22	31.608	0.15	2,3-Dimethylpyrazine
23	33.401	0.28	4,5-Dimethylthiazole
24	33.632	0.11	Dimethyl trisulfide
25	34.195	2.23	2-Ethyl-6-methylpyrazine
26	35.424	0.68	Trimethylpyrazine
27	35.893	0.12	2-Methyl-5-isopropyl pyrazine
28	36.242	0.19	• 2-Propylpyrazine
29	37.340	0.40	2,6-Diethylpyrazine
30	38.032	0.28	2,5-Dimethyl-3-ethylpyrazine
31	38.248	0.77	3-Methylthiopropanol
32	38.735	0.23	2-Furancarboxaldehyde
33	39.083	0.30	5-Ethyl-2,3-dimethylpyrazine
34	39.269	0.68	2-Methyl-5-propylpyrazine
35	39.942	0.28	Tetramethylpyrazine
36	40.602	0.10	Acetic acid
37	41.295	0.55	Decanal
38	41.370	0.72	1-(2-Furanyl) ethanone
39	41.776	0.32	Tetradecamethylcycloheptasiloxane
40	42.458	9.94	Benzaldehyde

41	44.312	0.70	L-Linalool
42	45.031	0.08	3,5,5-Trimethylhexylamine
43	47.823	0.17	4-Oxopentanoic acid ethyl ester
44	48.019	0.48	2-Acetyl-5-methylfuran
45	48.466	0.15	Methyl benzoate
46	48.710	0.20	1-(2-Pyrazinyl) ethanone
47	48.837	0.24	2-Isoamyl-6-methylpyrazine
48	49.068	0.14	*Cis*-3-Methyl-2-*n*-propylthiophane
49	49.551	2.48	Benzeneacetaldehyde
50	49.750	1.85	Ethyl decanoate
51	50.054	0.30	1-Phenyl-ethanone
52	50.713	0.75	2-Furanmethanol
53	50.911	0.11	2,5-Dimethyl-3-(3-methylbutyl)pyrazine
54	51.362	0.53	1-Chlorododecane
55	51.580	0.26	Hexadecamethylcyclooctasiloxane
56	51.946	1.19	4,5,6,7-Tetrahydro-2-benzofuran-1(3H)-one
57	52.274	0.26	2-Methylmercaptomethylbut-2-enal
58	52.547	0.25	l-(6-Methyl-2-pyrazinyl)-1-ethanon
59	52.677	1.04	2-Acetyl-4-methy1thiazole
60	53.062	0.88	2-(4-Methyl-3-cyclohexen-1-yl)-2-propanol
61	53.741	1.51	Dodecanal
62	54.019	0.32	3-Methylthiopropanol
63	54.243	0.10	1(3H)-Isobenzofuranone
64	54.392	0.39	1-Phenylpropan-2-one
65	5.696	0.26	1-Methyl-2-fluoro-4,5-dicyanoimidazole
66	54.792	0.18	1-Amido-1-cyano-3-methylbut-1-ene
67	55.663	0.16	2-Amino-4,6-dimethoxypyrimidine
68	56.381	0.57	1-(4-Methyl-2-thienyl) ethanone
69	56.705	0.14	1-(3-Thienyl) ethanone
70	56.854	0.09	4-Ethyl-5-methylthiazole
71	57.013	0.22	4-Methoxy-5-ethylimidazole
72	57.745	0.22	5-Methyl-5-hydroxy-1,2-octadien-7-yn-4-one
73	58.234	0.18	2-Chlorobenzaldehyde
74	58.371	0.51	2-Phenylpropenal
75	58.593	0.16	1-Phenyl-2-butanone
76	58.87	0.88	5-Acetyl-2,4-dimethylthiazole
77	59.858	0.24	4-Propylbenzaldehyde
78	60.383	0.16	2-Methyl-5-propylthiophene
79	61.082	6.43	Ethyl dodecanoate
80	61.208	1.46	3-Methylcinnoline
81	61.534	0.42	2-Methoxyphenol
82	61.945	0.24	4-Methoxybenzenethiol
83	62.503	0.11	Benzenemethanol
84	64.343	3.49	Benzeneethanol
85	64.874	0.29	1,2-Dithian-4-one
86	67.263	1.26	Acetylpyrrole
87	67.430	3.87	1-Decene
88	68.046	0.68	2-Phenylbutan-1-ol
89	68.412	0.21	4(1H)-Quinazolinone
90	68.901	0.68	Phenol
91	70.041	0.25	1,3-Cyclopentanedione, 2-(2,4,6-cycloheptatrien-1-yl)
92	70.154	0.74	4-Ethylguaiacol
93	71.432	0.94	Ethyl tetradecanoate
94	72.377	0.94	5-Methyl -2-phenyl -2-hexenal
95	73.969	1.51	Octanoic acid
96	77.225	0.27	Methyl 4,6-decadieny1 ether
97	80.165	0.19	3-Phenylpyridine
98	80.934	0.77	Ethyl hexadecanoate
99	82.475	0.34	Dimethyl phthalate
100	83.195	0.31	2,4-Di-tert-butylphenol
101	83.409	2.26	*n*-Decanoic acid

102	83.523	3.81	Decanoic acid
103	92.111	9.61	Dodecanoic acid
